# Supplementary material for: Active versus restrictive approach to isolated hypotension in preterm neonates: A Systematic Review, Meta-analysis and GRADE based Clinical Practice Guideline
Source: PLoS One. 2025 Mar 18;20(3):e0309520. doi: 10.1371/journal.pone.0309520 (PMC11918419; doi:10.1371/journal.pone.0309520)
Supplement: S1 File — S1 Table. Literature search strategy. S2 Table. Modified GRADE approach for reporting of results of the systematic review. S3 Table. Risk of bias assessment of the included studies. S4 Table. Evidence to Decision framework. Appendix Narrative review of included studies. S5 Table: Data extraction table. (PDF) [file pone.0309520.s001.pdf]

Supplement file

S1a Table: Literature search strategy

S1b Table: Updated literature search strategy

S2 Table: Modified GRADE approach for reporting of results of the systematic review

S3 Table: Risk of bias assessment of the included studies

S4 Table: Evidence to Decision framework

Appendix: Narrative review of included studies

S5 Table: Data extraction table

S1a Table: Literature search strategy

Database(s): **Ovid MEDLINE(R) ALL and EMBASE** 1946 to July 15, 2023

	**Searches**	**Medline**	**Embase**
1	exp infant, large for gestational age/ or exp infant, low birth weight/ or exp infant, postmature/ or infant, premature/ or exp infant, extremely premature/	91012	93032
2	(premie or preterm* or premature or "Low birth weight" or lbw or vlbw or elbw or "Low birth weights" or "Low birthweight" or "Low birthweights" or Infant* or "Small gestational age" or SGA or "Extremely premature").ab,ti.	608553	650085
3	1 or 2	623914	634575
4	exp Hypotension/	29798	30378
5	"cardiovascular insufficienc*".ab,ti.	237	267
6	mean blood pressure.ab,ti.	8071	8172
7	(4 or 5 or 6) and (1 or 2)	1038	1126

S1b Table: Updated literature search strategy

Database(s): **Ovid MEDLINE(R) ALL** 16th July to 1st April, 2024

#	Searches	Results
1	exp infant, large for gestational age/ or exp infant, low birth weight/ or exp infant, postmature/ or infant, premature/ or exp infant, extremely premature/	94279
2	(premie or preterm* or premature or "Low birth weight" or lbw or vlbw or elbw or "Low birth weights" or "Low birthweight" or "Low birthweights" or Infant* or "Small gestational age" or SGA or "Extremely premature").ab,ti.	638983
3	1 or 2	654568
4	exp Hypotension/	30588
5	"cardiovascular insufficienc*".ab,ti.	242
6	mean blood pressure.ab,ti.	8285
7	(4 or 5 or 6) and (1 or 2)	1072
8	limit 7 to dt=20230216-20241231 (Search updated from 16th July 2023)	28

Database(s): **Embase Classic+Embase** 16th July to 1st April, 2024

#	Searches	Results
1	exp infant, large for gestational age/ or exp infant, low birth weight/ or exp infant, postmature/ or infant, premature/ or exp infant, extremely premature/	188207
2	(premie or preterm* or premature or "Low birth weight" or lbw or vlbw or elbw or "Low birth weights" or "Low birthweight" or "Low birthweights" or Infant* or "Small gestational age" or SGA or "Extremely premature").ab,ti.	869138
3	1 or 2	908078
4	exp Hypotension/	193647
5	"cardiovascular insufficienc*".ab,ti.	399
6	mean blood pressure.ab,ti.	11834
7	(4 or 5 or 6) and (1 or 2)	6329
8	limit 7 to dc (Search updated from 16th July 2023)	447
9	limit 8 to "remove medline records"	227

Database: Web of Science Core Collection Inception to 1st April, 2024

#	Search Query	Results
1	(premie or preterm* or premature or "Low birth weight" or lbw or vlbw or elbw or "Low birth weights" or "Low birthweight" or "Low birthweights" or Infant* or "Small gestational age" or SGA or "Extremely premature") (Title) AND (premie or preterm* or premature or "Low birth weight" or lbw or vlbw or elbw or "Low birth weights" or "Low birthweight" or "Low birthweights" or Infant* or "Small gestational age" or SGA or "Extremely premature") (Abstract) AND (premie or preterm* or premature or "Low birth weight" or lbw or vlbw or elbw or "Low birth weights" or "Low birthweight" or "Low birthweights" or Infant* or "Small gestational age" or SGA or "Extremely premature") (Author Keywords)	62466
2	Hypotension (Title) AND Hypotension (Abstract) AND Hypotension (Author Keywords)	3409
3	(cardiovascular NEAR/2 insufficienc*) or ((mean or average) NEAR/2 "blood pressure") (Title) AND (cardiovascular NEAR/2 insufficienc*) or ((mean or average) NEAR/2 "blood pressure") (Abstract) AND (cardiovascular NEAR/2 insufficienc*) or ((mean or average) NEAR/2 "blood pressure") (Author Keywords)	35
4	low blood pressure (Title) AND low blood pressure (Abstract) AND low blood pressure (Author Keywords)	114
5	#2 OR #3 OR #4	3557
6	#5 AND #1	51

S2 Table: Modified GRADE approach for reporting of results of the systematic review

Descriptor	Criteria
Clinical benefit/harm	Statistically significant result<br>High certainty evidence<br>Biological mechanism(s) well established<br>Point estimates of underlying studies are consistently in one direction<br>Optimal information size reached[6]
Probable clinical benefit/harm	Statistically significant result<br>Moderate or high certainty evidence<br>Evidence of biological plausibility<br>Point estimates of underlying studies are predominatly in one direction<br>Close to optimal information size or summary confidence interval is sufficiently narrow to give confidence that the true effect would be clinically meaningful if it is only in the ballpark of the summary estimate.
Possible clinical benefit/harm	Statistically significant result<br>Low or very low certainty evidence<br>Few studies, wide summary confidence interval or effect is driven by one or two heavily weighted studies
Improbable benefit/harm	Statistically non-significant result<br>Moderate or high certainty evidence<br>Point estimates of underlying studies are close to and on both sides of the line of null effect
No clinical benefit/harm	Statistically non-significant result<br>High certainty evidence<br>Point estimates of underlying studies are close to and on either side of the line of null effect<br>Majority of underlying studies are adequately powered for outcome of interest<br>Optimal information size reached reached
Clinical benefit/harm cannot be excluded	Statistically non-significant result<br>Low or very low certainty evidence<br>Few studies<br>Wide confidence intervals

S3 Table: Risk of bias assessment of the included studies **RCTs**

Study	Randomization process	Deviations from intended interventions	Missing outcome data	Measurement of the outcome	Selection of the reported result	Overall bias
**Batton 2012**	Low	High	Low	Low	Low	High
**HIP 2021**	Low	High	Low	Low	Low	High
**Pereira 2019**	Low	Low	Low	Low	Low	Low

Non-RCTs

Study	Confounding	Selection	Classification of interventions	Deviation from intended intervention	Missing data	Measurement of outcomes	Selective reporting	Overall
**Aladangady 2023**	Moderate	Moderate	Serious	NI	Low	Low	Moderate	Serious
**Bada 1990**	Serious	Low	Low	Low	Low	Low	Moderate	Serious
**Batton 2009**	Moderate	Serious	Serious	NI	Low	Low	Low	Serious
**Batton 2013**	Moderate	Moderate	Moderate	NI	Low	Low	Low	Moderate
**Batton 2014**	Moderate	Moderate	Moderate	NI	Low	Low	Low	Moderate
**Batton 2016**	Moderate	Moderate	Moderate	NI	Moderate	NI	Moderate	Moderate
**Binder-Heschl 2017**	Moderate	Moderate	Low	Low	Low	Serious	Moderate	Moderate
**Carrapato 2018**	Serious	Moderate	Serious	Ni	Low	Low	Low	Serious
**Cunningham 1999**	Serious	Moderate	Serious	NI	Low	Low	Low	Serious

**Dempsey 2009**	Serious	Moderate	Serious	NI	Low	Low	Low	Serious
**Deshpande 2023**	Moderate	Moderate	Low	Low	Low	Serious	Moderate	Moderate
**Dammann 2002**	Serious	Moderate	Serious	NI	Low	Low	Low	Serious
**D'Souza 1995**	Serious	Low	Moderate	Low	Low	Low	Moderate	Serious
**Durrmeyer 2017**	Moderate	Moderate	Moderate	NI	Low	Low	Low	Moderate
**Durmeyer 2017**	Moderate	Low	Moderate	NI	Moderate	Low	Low	Moderate
**Fanaroff 2006**	Moderate	NI	Serious	NI	Low	Low	Low	Serious
**Faust 2015**	Moderate	Low	Moderate	NI	Moderate	Low	Low	Moderate
**Fernandez 2015**	Moderate	Low	Serious	NI	Low	Low	Low	Moderate
**Gogcu 2020**	Serious	Moderate	Serious	NI	Low	Low	Low	Serious
**Gronlund 1994**	Serious	Low	Low	Low	Low	Low	Moderate	Serious
**Kim 2018**	Serious	Low	Serious	NI	Serious	Low	Moderate	Serious
**Kiss 2023**	NA	Serious	Serious	Serious	Moderate	Serious	Moderate	Serious
**Kuint 2008**	Serious	Moderate	Serious	NI	Low	Low	Moderate	Serious
**Lee 1999**	Moderate	Moderate	Low	Low	Low	Low	Moderate	Moderate
**Leflore 2000**	Moderate	Serious	Serious	NI	Low	Low	Moderate	Serious

**Liping 2023**	Serious	NI	Serious	NI	Low	Serious	Moderate	Serious
**Liu 2022**	Moderate	Moderate	Moderate	NI	Low	Low	Moderate	Moderate
**Limperopoulos 2007**	Moderate	Low	Low	Low	Low	Low	Moderate	Moderate
**Logan 2011**	Moderate	Low	Moderate	NI	Low	Low	Low	Moderate
**Martens 2003**	Moderate	Low	Moderate	NI	Low	Low	Low	Moderate
**Meek 1999**	NI	Low	Low	Low	Low	Low	Moderate	Moderate
**Miall-Allen 1987**	Critical	Low	Moderate	NI	Low	Low	Moderate	Critical
**Peter 2017**	Moderate	Low	Serious	NI	Low	Low	Low	Moderate
**Pellicer 2009**	Moderate	Low	Moderate	NI	Low	Low	Low	Moderate
**Trounce 1988**	NI	Low	Serious	Low	Low	Low	Moderate	Moderate
**Victor 2006**	Moderate	Low	Moderate	Low	Low	Low	Low	Moderate
**So Yoon Ahn 2011**	Moderate	Low	Serious	NI	Low	Low	Serious	Serious
**Watkins 1998**	Moderate	Moderate	Serious	NI	Low	Low	Moderate	Serious
**Weindling 1985**	Serious	Low	Low	Low	Low	Low	Moderate	Serious

S4 Table: Evidence to Decision framework

**Should active treatment (isolated hypotension without any clinical or biochemical signs of poor perfusion) vs. restrictive treatment (no treatment of isolated hypotension or treatment of hypotension with clinical or biochemical signs of poor perfusion) be used for preterm neonates born at less than 37 weeks' gestation in the first week of life?**	
**POPULATION:**	Preterm neonates born at less than 37 weeks' gestation in the first week of life
**INTERVENTION:**	Active treatment (isolated hypotension without any clinical or biochemical signs of poor perfusion)
**COMPARISON:**	Restrictive treatment (no treatment of isolated hypotension or treatment of hypotension with clinical or biochemical signs of poor perfusion)
**MAIN OUTCOMES:**	Neonatal mortality (RCTs); Neonatal mortality (non-RCTs); Severe brain injury (defined as IVH > grade 2 or cystic PVL) (RCTs); Severe brain injury (defined as IVH > grade 2 or cystic PVL) (non-RCTs); NEC ≥ stage 2 (RCTs); NEC ≥ stage 2 (non-RCTs); PDA requiring treatment (RCTs); PDA requiring treatment (non RCT); Moderate to severe BPD (O2 or respiratory support requirement at 36 weeks' PMA) (RCTs); Moderate to severe BPD (O2 / respiratory support requirement at 36 weeks' PMA (non-RCTs); Severe ROP (non-RCT); Mortality or neurodevelopmental impairment at 18-22 months' corrected age (non-RCT); Sensineural hearing loss at 12-24 months' corrected age (non-RCT)

ASSESSMENT

Problem

Is the problem a priority?

JUDGEMENT	RESEARCH EVIDENCE	ADDITIONAL CONSIDERATIONS
○ No<br>○ Probably no<br>○ Probably yes<br>● Yes<br>○ Varies<br>○ Don't know	Isolated hypotension in the initial days (especially within the 72 h) is common in very preterm (VPT) and extremely low gestational age neonates (ELGANs). (1, 2) Also, the definition of hypotension based on blood pressure (BP) values is a highly debated topic.(3) Further, there is paucity of randomised controlled trials (RCTs) comparing active treatment (isolated hypotension without any clinical or biochemical signs of poor perfusion) vs. restrictive treatment (Clinical signs of poor perfusion with or without low blood pressure) in VPT and ELGANs. However, multiple observational studies have explored this PICO. (4, 5, 6, 7, 8, 9, 10, 11) The results of these studies are differing, and hence debatable. Henceforth, it is imperative that the published literature on this subject be evaluated through a validated process to guide safe clinical practice and improve neonatal outcomes.	**Definitions of isolated hypotension evaluated in the studies**<br>The definitions for treatment of hypotension varied widely between these studies. The most commonly used definitions were mean arterial blood pressure (MAP) < gestational age (GA) in (weeks)(w) (12, 8, 13, 1, 2, 6, 5), MAP less than a particular centile value, (14, 15, 16, 7), MAP ≤ 25 mm Hg for > 2-3 consecutive readings (17, 3) and MAP < 30 mm Hg (10, 18, 19). Some of the studies had not taken into account BP values and relied on clinical / biochemical signs of poor perfusion alone as well. (20, 21)<br>**Comparison of different definitions to define hypotension**<br>Batton (2013) et al. in their prospective cohort study had compared 15 different definitions based on systolic blood pressure, diastolic blood pressure or MAP < 5th centile for ≥ 3 consecutive values, MAP ≤ 25 mm Hg for ≥ 3 consecutive values and MAP ≤ GA (w) in preterm neonates of < 26 w and of postnatal age < 24 hours (h). The authors concluded that neonatal outcomes did not improve with anti-hypotensive therapy for any of the 15 definitions and that a numeric cut-off alone for initiating anti-hypotensive therapy may not be utilized as outcomes were similar or worser in the treated group vs. the not treated group(3).<br>**MAP < GA (w) vs. MAP < 30 mm Hg**<br>Liu (2022) et al. in their prospective study of VPT neonates of < 72 h of age compared these two definitions of hypotension. Based on multivariate logistic regression, the authors concluded that hypotension based on either of the definitions was not an independent risk factor for poor prognosis (P>0.05)(22).<br>Limperopoulos (2007) et al. in their prospective study of preterm neonates of ≤ 30 w and < 72 h of age evaluated 3 definitions of hypotension: MAP < 30 mm Hg, MAP < GA (w) and MAP < 10th percentile of MAP for birth weight (BW) and postnatal age based on published normative data. The authors concluded that these definitions of hypotension did not predict any grade intraventricular hemorrhage (IVH)(23).<br>So Yoon Ahn (2011) et al. in their retrospective study of extremely low birth weight (ELBW) neonates of < 72 h of age compared 3 groups: a normotensive (N) group with no signs of poor perfusion, treatment group (T) group with MAP < GA (w) and received interventions including volume expansion, vasopressors or corticosteroids and permissive Group (P) group; MAP < GA (w) with signs of good perfusion and no intervention.

		After adjusting for baseline sickness, mortality [aOR: 3.30 (1.30 – 8.50)], IVH > stage 2 [aOR: 7.40 (2.60 – 21.50)] and bronchopulmonary dysplasia (BPD) (O2 requirement at 36 weeks' postmenstrual age (PMA)) [aOR: 3.60 (1.30 – 10.30)] were significantly higher in the T group when compared to N group. NEC ≥ stage 2, retinopathy of prematurity (ROP) requiring treatment and periventricular leukomalacia (PVL) were comparable between the P and N groups. No comparison adjusted for baseline sickness was evaluated for T vs. P group. The authors concluded that close follow up of ELBW neonates with isolated hypotension with no signs / symptoms of poor perfusion, and without intervention could prevent unnecessary use of inotropes and may result in neurological outcomes similar to neonates with normotension(8).

Desirable Effects

How substantial are the desirable anticipated effects?

JUDGEMENT	RESEARCH EVIDENCE	ADDITIONAL CONSIDERATIONS
● Trivial<br>○ Small<br>○ Moderate<br>○ Large<br>○ Varies<br>○ Don't know	**RCTs**<br>There were only 2 randomised controlled trials (RCTs) by Dempsey (2020) et al. and Pereira (2019) et al. that had compared active versus restrictive management of hypotension in VPT neonates. (1, 2) The effect estimates for the various critical and important outcomes from the meta-analysis of these RCTs were statistically non-significant (absolute effects not indicative of any direction). Batton (2012) et al. in their pilot RCT had indicated the various difficulties associated with conducting a RCT on this PICO. (24)<br>**Observational studies evaluating short term outcomes**<br>Meta-analysis of 2 observational studies enrolling preterm neonates of <29 w GA within 72 h showed that active treatment group had a lower risk of major brain injury (MBI) (IVH > grade or PVL) (Absolute risk reduction: 48 per 1000; 66 fewer to 15 fewer) and necrotising enterocolitis stage (NEC) ≥ 2 (Absolute risk reduction: 60 per 1000; 94 fewer to 12 fewer) when compared to restrictive treatment group.(9, 10) No study had evaluated the outcome of NEC ≥ stage 2 for VPT neonates of less than 24 h of life.	

Undesirable Effects

How substantial are the undesirable anticipated effects?

JUDGEMENT	RESEARCH EVIDENCE	ADDITIONAL CONSIDERATIONS

○ Trivial<br>● Small<br>○ Moderate<br>○ Large<br>○ Varies<br>○ Don't know	**Observational studies evaluating short term outcomes**<br>As stated before, the evidence from RCTs is inconclusive and clinical benefit or harm could not be ruled out for any of the undesirable effects. However, the results from the observational studies indicate that active treatment of hypotension might possibly increase the risk of MBI (Absolute risk: 65 per 1000; 28 more to 108 more), PDA requiring treatment (Absolute risk: 77 per 1000; 16 more to 155 more), composite outcome of mortality or neurodevelmental impairment (NDI) at 18-22 months' CA (Absolute risk: 138 per 1000; 20 more to 267 more) and sensineural hearing loss at 2 years' CA (Case control study, aOR (adjusted odds ratio): 3.60; 95% CI (confidence interval) 1.30-9.70), absolute risk not calculable). Since the lower 95% CI of the absolute risk was < 5 % for all of the important and critical outcomes, the magnitude of undesirable effects was adjudged as small.<br>There was significant heterogeneity in the meta-analysis for the outcome of MBI. Hence to explore the cause of heterogeneity, sub-group analyses were performed. Sub-group analysis based on the postnatal age of the enrolled neonates revealed that active treatment of hypotension in neonates of < 24 h was life was associated with increased risk of MBI. However, meta-analysis of those studies that had enrolled neonates of < 72 h showed that active treatment of hypotension might possibly decrease the risk of MBI. This sub-group difference was not found for the other outcomes evaluated.<br>**Observational studies that had evaluated long term critical outcomes**<br>There were 3 observational studies that had evaluated the composite outcome of mortality or NDI (critical outcome)(8, 17, 4). In these studies, the restrictive treatment group with isolated hypotension was compared with a group of neonates with no hypotension. Of these studies, 2 studies [Batton (2009) et al.. and Logan (2011) et al.] which had evaluated extremely low gestational age neonates (ELGANs) of < 24 h of postnatal age had shown that neonates treated with restrictive approach may possibly have similar long term neurodevelopmental outcomes (NDO) when compared to those neonates who did not have hypotension. Another retrospective cohort study by Ahn (2011) et al. which had enrolled ELBW neonates of < 72 h had indicated that long term NDI was comparable between the restrictive treatment group and those with no hypotension at all. (8) To summarise, most of the studies reporting on long term outcomes of mortality or NDI and NDI alone had shown that restrictive treatment group might have similar outcomes when compared to normal neonates.	Peter (2017) et al. in their retrospective cohort study of preterm neonates <29 w and < 72 h of age in their multi-variate regression analysis indicated that isolated hypotension was not associated with the combined outcome of mortality or severe IVH. However, only GA and vasopressor support (initiated based on MAP cut-off with clinical signs of poor perfusion) were predictors of the composite outcome (29).<br>Ahn (2011) et al. in their retrospective study of ELBW neonates of < 72 h of age compared 3 groups: a normotensive (N) group with no signs of poor perfusion, treatment group (T) group with MAP < GA and received intervention including volume expansion, vasopressors or corticosteroids and permissive Group (P) group; MAP < GA with signs of good perfusion and no intervention. After adjusting for baseline sickness, mortality [aOR: 3.30 (1.30 – 8.50)], IVH > stage 2 [aOR: 7.40 (2.60 – 21.50)] and BPD (O2 requirement at 36 weeks' PMA) [aOR: 3.60 (1.30 – 10.30)] were significantly higher in the T group when compared to N group. NEC ≥ stage 2, ROP requiring treatment and PVL were comparable between the P and N groups. On the contrary, the P group had comparable outcomes to N group. No comparison adjusted for baseline sickness was calculated for T vs. P group. The authors concluded that close follow up of ELBW neonates with isolated hypotension with no signs / symptoms of poor perfusion, and without intervention could prevent unnecessary use of inotropes and may result in neurological outcomes similar to neonates with normotension(8).<br>Martens (2003) et al. in their prospective study concluded that isolated hypotension defined as MAP < 30 mm Hg on at least 2 occasions in preterm neonates (< 32 w, any postnatal age) was not associated with abnormal neurological examination at term equivalent gestation(19).<br>Fernandez (2015) et al. compared 4 different definitions of hypotension of which one was MAP < GA without any signs of poor perfusion in two sub-groups of neonates: term and preterm neonates of < 72 h of postnatal age. The authors reported that MAP < GA was not associated with mortality, but was associated with greater number of days on mechanical ventilation and oxygen support(12).<br>Carrapato (2018) et al. in their retrospective study reported that in preterm neonates of < 32 w GA and < 72 h of postnatal age; respiratory distress syndrome (RDS), sepsis and anemia were significantly associated with clinical hypotension irrespective of BP readings(13). IVH and PDA had significant association with GA and BW, independent of isolated hypotension or clinical hypotension. PVL was significantly associated with clinical hypotension with or without low MAP, and not with low MAP alone. Authors concluded that if persistent hypotension is to be treated, rather than treating low MAP in isolation, clinical signs of hypotension should guide treatment and that permissive hypotension especially if transient, in the absence of clinical signs of hypoperfusion may be considered.<br>Lee (2012) et al. in their retrospective cohort study of preterm neonates of <29 w and < 7 d reported that isolated hypotension defined as MAP < GA was not associated with poor short term outcomes or mortality.<br>Batton (2014) et al. concluded that BP increases spontaneously over the first 24 h in preterm neonates of <27 w and that the rate of rise in BP was similar between treated vs. untreated neonates(30).<br>Watkins (1998) et al. in their retrospective cohort study of preterm neonates of ≤ 34 weeks' gestation and whose invasive blood pressure (IBP) were monitored until 96 h of postnatal age reported in their unadjusted analysis that hypotension (defined as MAP < 10th centile for GA, BW and postnatal age for > 2 consecutive readings) was associated with any grade IVH and that hypotension episodes were observed on the day before as well the day after the occurrence of IVH. The authors concluded that IVH may have given rise to the hypotension or the vice versa(14).<br>Bada (1990) et al. in their prospective observational study of VPT neonates reported that those neonates with severe grade IVH had a greater percentage of time with a coefficient of variation of

		MAP > 13% or <3% in the first 48 h when compared to their matched control subjects(31). Further, in the sub-group of ELBW neonates who developed severe grade IVH, the MAP was observed to be consistently low in the first 48 h of postnatal life. This study did not mention the protocol to define or treat hypotension.<br>D'Souza (1995) et al. in their prospective observational study of VPT neonates of ≤ 10 d of postnatal age reported that the coefficient of variation of the MAP values was higher in neonates on the day of IVH, and a similar trend was apparent on the day before(20).<br>Meek (1999) et al. in their prospective study of VPT neonates reported that those neonates with lowest cerebral blood flow (CBF) [5.8 (3.2–7.5) ml/100 g min)] in the first 24 h of life had severe IVH (> grade 2) when compared to those with a higher CBF [12.1 (6.1–24.7) ml/100 g/ min]. Further, the neonates with lowest CBF had significantly higher MAP in the first 24 h of life [35.0 (35–43) mm Hg] when compared to those with higher CBF [29.5 (25–40) mm Hg]. A reasonable inference that could be drawn from this finding is that increased MAP in the first 24 h of life might be associated with severe grade IVH(32).<br>Victor (2006) et al. in their prospective study of preterm neonates of < 30 w and postnatal age of < 48 h evaluated the effect of different levels of MAP on peripheral blood flow (PBF), cerebral fractional oxygenation (CFOE) using near infrared spectroscopy (NIRS) and cerebral electrical activity using aEEG. The authors reported that EEG became abnormal at MAP < 23 mm Hg, abnormally high CFOE was measured at 20 mm Hg, and PBF decreased at MAP range of 23-33 mm Hg. The authors concluded that cerebral perfusion is probably maintained at MAP levels above 23 mm Hg(15).<br>Cunningham (1999) et al. in their prospective observational study of very low birth weight (VLBW) neonates of ≤ 7 d of life reported that there was no significant association between the percentage of time the neonates' MAP was less than GA after adjusting for BW, GA; and IVH. The authors noted that there was a significant association with increased MAP variability and mortality in the first 7 days of life(25).<br>Grolund (1994) et al. in their prospective observational study of preterm neonates ≤ 36 w GA reported that elevated diastolic, mean and systolic blood pressure in the first 24 h of life were significantly associated with major brain injury in preterm newborn infants(26).<br>Trounce (1988) et al. in their retrospective cohort study of VLBW neonates concluded that there was no relation between hypotension or the development of PVL based on their multivariate regression analysis correcting for baseline sickness. The authors did not mention the details regarding the definition of hypotension, its management or the postnatal age till when these neonates were monitored(27).<br>Weindling (1985) et al. in their prospective cohort study of neonates (< 34 w and whose BPs were monitored till 5 days of life) based on their univariate regression analysis reported that there was no association between hypotension (MAP < 30 mm Hg on a single occasion or if signs of poor perfusion was present irrespective of MAP values) and PVL(18).<br>**Treatment of hypotension based on only clinical / biochemical signs as assessed by the clinician irrespective of BP readings**<br>Fanaroff (2006) et al. in their retrospective study of ELBW neonates of < 72 h of age evaluated treatment of hypoperfusion on the basis of associated vital signs, perfusion, and the overall clinical status of the neonate with no specific cut-off of MAP to define hypotension(28). The authors concluded that such an approach was significantly associated with delayed motor development, defined as a mental developmental index of < 70 after adjusting for baseline sickness. The authors emphasized the importance of establishing normative values for BP in ELBW infants.<br>MAP < 3rd percentile for GA or < 30 mm Hg with one clinical criteria of decreased urine output (<1 ml/kg/hour for >12 h) in VLBW neonates of < 7 d was evaluated by Kim (2018) et al., and the authors reported that after correcting for baseline sickness,

		hypotension in the first week along was associated with composite outcome of mortality or poor long term NDO (cognitive and language outcomes) as evaluated by BSID-III(16). This study may indicate that irrespective of definition used, a single clinical criterion to decide on anti-hypotensive therapy may not be appropriate in the restrictive group. Also, the clinical criteria used which was urine output < 1 ml/kg/hour for >12 h (relatively longer duration) in neonates in the first week of life might have resulted in delayed initiation of therapy for poor perfusion leading to poor outcomes.<br>Most of the aforementioned studies indicate that active approach treatment of isolated hypotension in preterm neonates may not be associated with additional benefits and some studies have also indicated possible harm with active treatment of hypotension. Some studies have indicated that increased MAP variability was associated poorer outcomes. There were a few studies that suggested that restrictive treatment based on a single clinical criteria along with a MAP cut off to define hypotension; and use of multiple clinical criteria without taking into account the MAP values may also result in harm. Hence it is important that restrictive treatment of hypotension be well defined. It is to be noted that there was wide variability between these studies in relation to the GA and postnatal age of the enrolled preterm neonates, the definition of hypotension and the time span during which these studies were conducted.

Certainty of evidence

What is the overall certainty of the evidence of effects?

JUDGEMENT	RESEARCH EVIDENCE	ADDITIONAL CONSIDERATIONS
○ Very low<br>● Low<br>○ Moderate<br>○ High<br>○ No included studies	The certainty of evidence for all the critical outcomes was low.	

Values

Is there important uncertainty about or variability in how much people value the main outcomes?

JUDGEMENT	RESEARCH EVIDENCE	ADDITIONAL CONSIDERATIONS
○ Important uncertainty or variability<br>○ Possibly important uncertainty or variability<br>● Probably no important uncertainty or variability<br>○ No important uncertainty or variability		The consensus of the working group was that there is probably no important uncertainty in how much the stakeholders value the main outcomes.

Balance of effects

Does the balance between desirable and undesirable effects favor the intervention or the comparison?

JUDGEMENT	RESEARCH EVIDENCE	ADDITIONAL CONSIDERATIONS

○ Favors the comparison<br>○ Probably favors the comparison<br>○ Does not favor either the intervention or the comparison<br>○ Probably favors the intervention<br>○ Favors the intervention<br>● Varies<br>○ Don't know	The conclusions derived from meta-analysis of observational studies predominantly indicate that restrictive treatment group was associated with better short- and long term-outcomes. However, sub-group analysis based on postnatal age of the neonates enrolled in these studies (<24 h vs. <72 h) indicated that restrictive treatment may be associated with poorer short term outcomes of MBI and NEC ≥ stage 2 in neonates of < 72 h.	It is to be noted that there was wide variability in the definitions used for "isolated hypotension". Further, the GA and the postnatal age also varied between the studies. Though majority of the studies have indicated that untreated isolated hypotension is not associated with poorer short- and long-term outcomes, few studies have indicated otherwise. Hence it is important to determine the lowest MAP below which perfusion to the vital organs and especially the brain is impaired if not treated.<br>Victor (2006) et al. in their prospective cohort study evaluated the effect of different levels of MAP on peripheral blood flow (PBF), cerebral fractional oxygenation (CFOE) using near infrared spectroscopy (NIRS) and cerebral electrical activity using aEEG (n=35). The unit practice was to maintain MAP > 10th centile, the attending physician was not a part of the research team. The EEG became abnormal at MAP < 23 mm Hg. Abnormally high CFOE was measured at 20 mm Hg. PBF decreased at MAP range of 23-33 mm Hg. The authors concluded that cerebral perfusion is probably maintained at MBP levels above 23 mm Hg.(15)

Resources required

How large are the resource requirements (costs)?"

JUDGEMENT	RESEARCH EVIDENCE	ADDITIONAL CONSIDERATIONS
○ Large costs<br>● Moderate costs<br>○ Negligible costs and savings<br>○ Moderate savings<br>○ Large savings<br>○ Varies<br>○ Don't know		Active treatment of isolated hypotension will require additional resources such as medications (normal saline, inotropes), infusion pumps and additional manpower for monitoring.

Certainty of evidence of required resources

What is the certainty of the evidence of resource requirements (costs)?

JUDGEMENT	RESEARCH EVIDENCE	ADDITIONAL CONSIDERATIONS
○ Very low<br>○ Low<br>○ Moderate<br>○ High<br>● No included studies	There were no included studies.	

Cost effectiveness

Does the cost-effectiveness of the intervention favor the intervention or the comparison?

JUDGEMENT	RESEARCH EVIDENCE	ADDITIONAL CONSIDERATIONS

○ Favors the comparison<br>● Probably favors the comparison<br>○ Does not favor either the intervention or the comparison<br>○ Probably favors the intervention<br>○ Favors the intervention<br>○ Varies<br>○ No included studies		Restrictive treatment of hypotension is shown to improve the short- and long-term outcomes. This might probably favor the restrictive treatment group in terms of cost effectiveness.

Equity
What would be the impact on health equity?

JUDGEMENT	RESEARCH EVIDENCE	ADDITIONAL CONSIDERATIONS
○ Reduced<br>● Probably reduced<br>○ Probably no impact<br>○ Probably increased<br>○ Increased<br>○ Varies<br>○ Don't know		By diverting valuable resources such as medication, equipment and manpower which could be utilized for other sicker neonates, active management of isolated hypotension might result in more inequity.

Acceptability
Is the intervention acceptable to key stakeholders?

JUDGEMENT	RESEARCH EVIDENCE	ADDITIONAL CONSIDERATIONS
○ No<br>● Probably no<br>○ Probably yes<br>○ Yes<br>○ Varies<br>○ Don't know	The research evidence predominantly indicate that active treatment of isolated hypotension might possibly be associated with poorer short- and long-term outcomes. These were critical and important outcomes. Further, the resources required would be more if an active approach to treat isolated hypotension is adopted. Considering all these, the guideline panel is of the view that the key stake holders may not accept the intervention (active treatment of hypotension).	

Feasibility
Is the intervention feasible to implement?

JUDGEMENT	RESEARCH EVIDENCE	ADDITIONAL CONSIDERATIONS
○ No<br>○ Probably no<br>● Probably yes<br>○ Yes<br>○ Varies<br>○ Don't know	The intervention includes administration of normal saline and / or use of inotropes. It also requires frequent monitoring of blood pressure and other vital signs. All these are available in level 3 NICUs and hence the intervention is probably feasible.	

SUMMARY OF JUDGEMENTS

	JUDGEMENT						
PROBLEM	No	Probably no	Probably yes	**Yes**		Varies	Don't know
DESIRABLE EFFECTS	**Trivial**	Small	Moderate	Large		Varies	Don't know
UNDESIRABLE EFFECTS	Trivial	**Small**	Moderate	Large		Varies	Don't know
CERTAINTY OF EVIDENCE	Very low	**Low**	Moderate	High			No included studies

	JUDGEMENT						
VALUES	Important uncertainty or variability	Possibly important uncertainty or variability	**Probably no important uncertainty or variability**	No important uncertainty or variability			
BALANCE OF EFFECTS	Favors the comparison	Probably favors the comparison	Does not favor either the intervention or the comparison	Probably favors the intervention	Favors the intervention	**Varies**	Don't know
RESOURCES REQUIRED	Large costs	**Moderate costs**	Negligible costs and savings	Moderate savings	Large savings	Varies	Don't know
CERTAINTY OF EVIDENCE OF REQUIRED RESOURCES	Very low	Low	Moderate	High			**No included studies**
COST EFFECTIVENESS	Favors the comparison	**Probably favors the comparison**	Does not favor either the intervention or the comparison	Probably favors the intervention	Favors the intervention	Varies	No included studies
EQUITY	Reduced	**Probably reduced**	Probably no impact	Probably increased	Increased	Varies	Don't know
ACCEPTABILITY	No	**Probably no**	Probably yes	Yes		Varies	Don't know
FEASIBILITY	No	Probably no	**Probably yes**	Yes		Varies	Don't know

TYPE OF RECOMMENDATION

Strong recommendation against the intervention	**Conditional recommendation against the intervention**	Conditional recommendation for either the intervention or the comparison	Conditional recommendation for the intervention	Strong recommendation for the intervention
○	●	○	○	○

CONCLUSIONS

Recommendation

- The guideline panel suggests not to treat isolated hypotension (hypotension without any clinical and / or biochemical signs of poor perfusion) in preterm neonates of less than 32 weeks' gestation in the first 24 hours of life (low certainty of evidence, weak recommendation).
- The mean arterial blood pressure cut-off to define hypotension may be centile based. The gestational age based centiles derived from the largest cohort of very preterm neonates from the German Neonatal Network may be used. Isolated hypotension with a mean arterial blood pressure value of less than 5 mm Hg (median mean arterial blood pressure - 5) for the corresponding gestational age may be treated (rescue therapy) in preterm neonates of less than 32 weeks' gestation in the first 24 hours of life. (Expert consensus)
- In preterm neonates of less than 32 weeks' gestation, isolated hypotension beyond the first 24 hours of life and within 72 hours of life may be treated. The mean arterial blood pressure cut-off for treatment of isolated hypotension beyond 24 hours of life may be a mean arterial blood pressure of less than the neonates' gestational age in weeks'. (low certainty of evidence, weak recommendation).
- There is insufficient evidence to recommend for or against treatment of isolated hypotension in preterm neonates of ≥ 32 weeks' gestation and in term neonates.

Justification

- Active treatment of isolated hypotension without any clinical or biochemical signs of hypoperfusion in preterm neonates of less than 32 weeks' gestation and in the first 24 hours of life was shown be associated with an increased risk of major brain injury (intraventricular hemorrhage > grade or periventricular leukomalacia), mortality or neurodevelopmental impairment at 18-22 months' corrected age and sensineural hearing loss at 2 years' corrected age. Apart from the results of the meta-analyses of these studies from which the conclusions were made, narrative review of many studies have also reported similar findings. Also, studies have shown that in very preterm neonates the mean arterial blood pressure rises physiologically over the first few days and more so in the first 24 hours of life. Actively treating a slower physiological rise in mean arterial blood pressure might result increased blood pressure variability which has also been shown to be associated with poorer short term outcomes. Further, studies on cerebral blood flow in very preterm neonates of < 24 hours of life have shown that cerebral perfusion is affected at a significantly lower mean arterial pressure, and also that cerebral perfusion could be compromised at relatively higher mean arterial blood pressure. These findings were also supported by some studies that had shown that increased variability of mean arterial blood pressure may be associated with major brain injury.
- Sub-group analyses indicated that active treatment of isolated hypotension in preterm neonates of less than 32 weeks' gestation and of postnatal age less than 72 hours was associated with decreased risk of necrotising enterocolitis ≥ stage 2 and major brain injury. Hence, separate recommendations were made based on the postnatal age.
- The largest cohort study (German Neonatal Network) has published nomograms of median mean arterial blood pressures for preterm neonates of less than 32 weeks' gestation and of less than 24 hours of postnatal age. There was a strong association between not treating isolated hypotension (when the cut-off mean arterial blood pressure used was median mean arterial pressure for the corresponding gestational age minus 5 mm Hg) and poor short-term outcomes. This cut-off was also used by the HIP trial in the placebo group as rescue therapy. Hence, this mean arterial blood pressure cut-off was chosen for rescue therapy for neonates with isolated hypotension in the first 24 hours of life.
- Since majority of the trials had assessed preterm neonates of less than 32 weeks', a separate recommendation could not be made for preterm neonates of ≥ 32 weeks' gestational age, and term neonates.
- The other aspects such as equity, cost-effectiveness, resource requirement, values, acceptability and feasibility were also taken into consideration before making this recommendation.

Subgroup considerations

- Sub-group analyses indicated that active treatment of isolated hypotension in preterm neonates of less than 32 weeks' gestation and of postnatal age less than 72 hours was associated with decreased risk of necrotising enterocolitis ≥ stage 2 and major brain injury.(10, 9) .Hence, separate recommendations were made based on the postnatal age of preterm neonates of less than 32 weeks' gestation (<24 h vs. < 72 h).
- The largest cohort study (German Neonatal Network) has published nomograms of median mean arterial blood pressures for preterm neonates of less than 32 weeks' gestation(7) .There was a strong association between not treating isolated hypotension (when the cut-off mean arterial blood pressure used was median mean arterial pressure for the corresponding gestational age minus 5 mm Hg) and poor short-term outcomes .(7, 9) This cut-off was also used by the HIP trial in the placebo group as rescue therapy. (1)Hence, this mean arterial blood pressure cut-off was suggested as the lowest mean arterial blood pressure for preterm neonates of less than 32 weeks and of <24 hours postnatal age with isolated hypotension in whom active treatment of isolated hypotension may be considered.

Implementation considerations

- The studies have used both invasive and non-invasive (oscillometry) methods for BP monitoring. If feasible invasive BP monitoring may. be preferred.(33)
- Preterm neonates who are treated by restrictive approach of not treating isolated hypotension should be closely monitored for clinical and / or biochemical signs of hypoperfusion such as tachycardia (>160-170 beats/min) (34), prolonged capillary refilling time (> 3-4 seconds) (35,36), low peripheral pulses, decreased urine output (< 1 ml/kg/ hour for 4 -6 hours; physiological oliguria or anuria should also be taken into account) (37), increasing lactate levels (> 3-4 mmol/L) (35) and base deficit (> 8 meq/L) (1,10). If feasible, functional echocardiography may be utilized as an adjunct to the clinical / biochemical criteria.(38)
- Warm shock may mimic isolated hypotension and hence those neonates at risk of early onset sepsis should be closely monitored as well.(39)
- If the decision to treat isolated hypotension is made, the target mean arterial blood pressure should be just more than or equal to the neonates' gestational age in weeks as higher mean arterial pressures and increased variability in blood pressures have been shown to be associated with major brain injury..

Monitoring and evaluation

- The short- and long-term outcomes of neonates who are managed as per these recommendations should be documented prospectively and the results should be evaluated.

Research priorities

- A large multi-centre randomized controlled trial comparing active treatment versus restrictive treatment of isolated hypotension in neonates of different gestational age cohorts is needed. As indicated by Batton (2012) et al. (24), low consent rates and lack of physician equipoise are the major barriers for conducting such a trial. Though Batton et al. suggested that such a trial may satisfy all the criteria for waiver of consent, this aspect should be thoroughly evaluated by the Ethical Committee of the respective centres.
- The approach to isolated hypotension in growth restricted neonates has not been studied and is a research priority.

- Nomograms for systolic, diastolic and mean blood pressure in very preterm neonates in the initial postnatal days from India is not available, making it a research priority.
- There is paucity of data on long-term neurodevelopmental outcomes of preterm neonates who are treated with a restrictive approach. This is another area of potential future research.

REFERENCES SUMMARY

1. Pereira SS, Sinha AK, Morris JK, Wertheim DF, Shah DK, Kempley ST et al. Blood pressure intervention levels in preterm infants: pilot randomized trial. Arch Dis Child Fetal Neonatal Ed. 2019 ;104(3):F298-F305.
2. Dempsey EM, Barrington KJ, Marlow N, O'Donnell CPF, Miletin J, Naulaers G, et al. Hypotension in Preterm Infants (HIP) randomized trial. Arch Dis Child Fetal Neonatal Ed. 2021 ;106(4):398-403.
3. Batton B, Li L, Newman NS, Das A, Watterberg KL, Yoder BA, Faix RG, Laughon MM, Stoll BJ, Van Meurs KP, Carlo WA, Poindexter BB, Bell EF, Sánchez PJ, Ehrenkranz RA, Goldberg RN, Laptook AR, Kennedy KA, Frantz ID 3rd, Shankaran S, et al.; Eunice Kennedy Shriver National Institute of Child Health & Human Development Neonatal Research Network. Use of antihypotensive therapies in extremely preterm infants. Pediatrics. 2013;131(6):e1865-73.
4. Logan JW, O'Shea TM, Allred EN, Laughon MM, Bose CL, Dammann O, et al; ELGAN Study Investigators. Early postnatal hypotension is not associated with indicators of white matter damage or cerebral palsy in extremely low gestational age newborns. J Perinatol. 2011 ;31(8):524-34.
5. Kuint J, Barak M, Morag I, Maayan-Metzger A. Early treated hypotension and outcome in very low birth weight infants. Neonatology. 2009;95(4):311-6.
6. Gogcu S, Washburn L, O'Shea TM. Treatment for hypotension in the first 24 postnatal hours and the risk of hearing loss among extremely low birth weight infants. J Perinatol. 2020 ;40(5):774-780.
7. Faust K, Härtel C, Preub M, Rabe H, Roll C, Emeis M, et al; Neocirculation project and the German Neonatal Network (GNN). Short-term outcome of very-low-birthweight infants with arterial hypotension in the first 24 h of life. Arch Dis Child Fetal Neonatal Ed. 2015;100(5):F388-92.
8. Ahn SY, Kim ES, Kim JK, Shin JH, Sung SI, Jung JM, et al. Permissive hypotension in extremely low birth weight infants (≤1000 gm). Yonsei Med J. 2012;53(4):765-71.
9. Durrmeyer X, Marchand-Martin L, Porcher R, Gascoin G, Roze JC, Storme L, et al; Hemodynamic EPIPAGE 2 Study Group. Abstention or intervention for isolated hypotension in the first 3 days of life in extremely preterm infants: association with short-term outcomes in the EPIPAGE 2 cohort study. Arch Dis Child Fetal Neonatal Ed. 2017 ;102(6):490-496.
10. Aladangady N, Sinha A, Banerjee J, et al. Comparison of clinical outcomes between active and permissive blood pressure management in extremely preterm infants. NIHR Open Res; 2023.
11. Dammann O, Allred EN, Kuban KC, Van Marter LJ, Pagano M, Sanocka U, Leviton A; Developmental Epidemiology Network. Systemic hypotension and white-matter damage in preterm infants. Dev Med Child Neurol. 2002;44(2):82-90.
12. Fernandez E, Watterberg KL, Faix RG, Yoder BA, Walsh MC, Lacy CB et al; Eunice Kennedy Shriver National Institute of Child Health and Human Development Neonatal Research Network. Definitions of cardiovascular insufficiency and relation to outcomes in critically ill newborn infants. Am J Perinatol. 2015 ;32(11):1024-30.
13. Carrapato MRG, Andrade T, Caldeira T. Hypotension in small preterms: what does it mean? J Matern Fetal Neonatal Med. 2019;32(23):4016-4021.
14. Watkins AMC, West CR, Cooke RWI. Blood pressure and cerebral hemorrhage and ischemia in very low birthweight infants. Early Hum Dev 1989; 19: 103.
15. Victor S, Marson AG, Appleton RE, Beirne M, Weindling AM. Relationship between blood pressure, cerebral electrical activity, cerebral fractional oxygen extraction, and peripheral blood flow in very low birth weight newborn infants. Pediatr Res. 2006 ;59(2):314-9.
16. Kim TH, Moon CJ, Sung IK, Youn YA. Hypotension within 1 week of life associated with poor short- and long-term outcomes in very low birth weight infants. Cardiol Young. 2018 ;28(8):1037-1041.
17. Batton B, Zhu X, Fanaroff J, Kirchner HL, Berlin S, Wilson-Costello D, et al. Blood pressure, anti-hypotensive therapy, and neurodevelopment in extremely preterm infants. J Pediatr. 2009;154(3):351-7, 357.e1.
18. Weindling AM, Wilkinson AR, Cook J, Calvert SA, Fok TF, Rochefort MJ. Perinatal events which precede periventricular hemorrhage and leukomalacia in the newborn. Br J Obstet Gynaecol 1985; 92(12): 1218 – 1223.
19. Martens SE, Rijken M, Stoelhorst GM, van Zwieten PH, Zwinderman AH, Wit JM, Hadders-Algra M, Veen S; Leiden Follow-Up Project on Prematurity, The Netherlands. Is hypotension a major risk factor for neurological morbidity at term age in very preterm infants? Early Hum Dev. 2003 Dec;75(1-2):79-89.
20. D'Souza SW, Janakova H, Minors D, Suri R, Waterhouse J, Appleton G et al. Blood pressure, heart rate, and skin temperature in preterm infants: associations with periventricular haemorrhage. Arch Dis Child Fetal Neonatal Ed 1995; 72(3): F162 – F167.
21. Fanaroff AA, Fanaroff JM. Short- and long-term consequences of hypotension in ELBW infants. Semin Perinatol. 2006 ;30(3):151-5.
22. Liu CH, Peng SC, Jin F, Xia SW. 低血压对胎龄<32 周早产儿近期预后的影响 [Influence of hypotension on the short-term prognosis of preterm infants with a gestational age of <32 weeks]. Zhongguo Dang Dai Er Ke Za Zhi. 2022;24(11):1195-1201.
23. Limperopoulos C, Bassan H, Kalish LA, Ringer SA, Eichenwald EC, Walter G et al. Current definitions of hypotension do not predict abnormal cranial ultrasound findings in preterm infants. Pediatrics. 2007;120(5):966-77.
24. Batton BJ, Li L, Newman NS, Das A, Watterberg KL, Yoder BA, Faix RG, Laughon MM, Van Meurs KP, Carlo WA, et al; Eunice Kennedy Shriver National Institute of Child Health and Human Development Neonatal Research Network. Feasibility study of early blood pressure management in extremely preterm infants. J Pediatr. 2012;161(1):65-9.e1.
25. Cunningham S, Symon AG, Elton RA, Zhu C, McIntosh N. Intra-arterial blood pressure reference ranges, death and morbidity in very low birthweight infants during the first seven days of life. Early Hum Dev. 1999;56(2-3):151-65.
26. Gronlund JU, Korvenranta H, Kero P, Jalonen J, Valimaki IA. Elevated arterial blood pressure is associated with peri-intraventricular hemorrhage. Eur J Pediatr 1994; 153(11): 836–841.
27. Trounce JQ, Shaw DE, Levene MI, Rutter N. Clinical risk factors and periventricular leukomalacia. Arch Dis Child 1988; 63: 17–22.
28. Fanaroff JM, Wilson-Costello DE, Newman NS, Montpetite MM, Fanaroff AA. Treated hypotension is associated with neonatal morbidity and hearing loss in extremely low birth weight infants. Pediatrics. 2006 ;117(4):1131-5.
29. St Peter D, Gandy C, Hoffman SB. Hypotension and Adverse Outcomes in Prematurity: Comparing Definitions. Neonatology. 2017;111(3):228-233.
30. Batton B, Li L, Newman NS, Das A, Watterberg KL, Yoder BA, Faix RG, et al; Eunice Kennedy Shriver National Institute of Child Health and Human Development Neonatal Research Network. Evolving blood pressure dynamics for extremely preterm infants. J Perinatol. 2014 ;34(4):301-5.
31. Bada HS, Korones SB, Perry EH, Arheart KL, Ray JD, Pourcyrous M et al. Mean arterial blood pressure changes in premature infants and those at risk for intraventricular hemorrhage. J Pediatr 1990; 117(4): 607–614.
32. Meek JH, Tyszczuk L, Elwell CE, Wyatt JS. Low cerebral blood flow is a risk factor for severe intraventricular hemorrhage. Arch Dis Child Fetal Neonatal Ed 1999; 81(1): F15 – F18.
33. Dionne JM, Bremner SA, Baygani SK, Batton B, Ergenekon E, Bhatt-Mehta V, et al.; International Neonatal Consortium. Method of Blood Pressure Measurement in Neonates and Infants: A Systematic Review and Analysis. J Pediatr. 2020 ;221:23-31.e5.
34. Davis AL, Carcillo JA, Aneja RK, Deymann AJ, Lin JC, Nguyen TC et al. American College of Critical Care Medicine Clinical Practice Parameters for Hemodynamic Support of Pediatric and Neonatal Septic Shock. Crit Care Med. 2017 ;45(6):1061-1093.
35. Cady LD Jr, Weil MH, Afifi AA, Michaels SF, Liu VY, Shubin H. Quantitation of severity of critical illness with special reference to blood lactate. Crit Care Med (1973) 1(2):75–80.
36. Osborn DA, Evans N, Kluckow M. Clinical detection of low upper body blood flow in very premature infants using blood pressure, capillary refill time, and central-peripheral temperature difference. Arch Dis Child Fetal Neonatal Ed. 2004. 89(2):F168–73.

37. Zappitelli M, Ambalavanan N, Askenazi DJ, Moxey-Mims MM, Kimmel PL, Star RA et al. Developing a neonatal acute kidney injury research definition: a report from the NIDDK neonatal AKI workshop. Pediatr Res. 2017;82(4):569-573.
38. de Boode WP, van der Lee R, Horsberg Eriksen B, Nestaas E, Dempsey E, Singh Y, et al.; European Special Interest Group 'Neonatologist Performed Echocardiography' (NPE). The role of Neonatologist Performed Echocardiography in the assessment and management of neonatal shock. Pediatr Res. 2018;84(Suppl 1):57-67.
39. Brierley J, Carcillo JA, Choong K, Cornell T, Decaen A, Deymann A et al. Clinical practice parameters for hemodynamic support of pediatric and neonatal septic shock: 2007 update from the American College of Critical Care Medicine. Crit Care Med. 2009;37(2):666-88.

Appendix: Narrative review of included studies

Studies on association between IH and short-term outcomes

Batton et al. in their prospective cohort study had compared 15 different definitions based on systolic blood pressure, diastolic blood pressure or MAP (< 5th centile), MAP ≤ 25 mm Hg and MAP ≤ GA (w) for ≥ 3 consecutive values in ELGANs of < 26 weeks and of postnatal age < 24 hours. The authors concluded that a numeric cut-off alone for initiating anti-hypotensive therapy may not translate to better outcomes. (1) Similar conclusions were made by Lee et al. and Kiss et al. (2,3) Ahn et al., Batton et al., Cunningham et al., Lee et al., Liu et al., Limperopoulos et al., Martens et al., Peter et al., Trounce et al. and Weindling et al. did not report any association between untreated IH in VPTI and ELGANs in the first few days of postnatal life and IVH, severe ROP, NEC ≥ stage 2, PVL or mortality. (4–13). On the contrary, Liping et al. in their retrospective study on ELGANs of less than 24 hours of age reported that hypotensive infants had significantly higher risk of morbidities including PDA requiring treatment, IVH > grade 2, pulmonary hemorrhage and mortality within the first 7 days of life. However, the control group of infants were normotensive, less sicker and the results were not adjusted for baseline sickness. (14)

Fernandez et al. who had evaluated both term and preterm neonates concluded that IH was not associated with mortality, but with increased days of mechanical ventilation and oxygen requirement. (15) Watkins et al. in their retrospective cohort study of preterm neonates of ≤ 34 weeks who were monitored until 96 hours of age reported in their unadjusted analysis that IH was associated with any grade IVH and that hypotension episodes were observed on the day before as well the day after the occurrence of IVH.(16) The authors concluded that IVH may have given rise to the hypotension or the vice versa. These were the two studies that had found some association between IH and poorer outcomes.

Miall-Allen et al. in their study of VPTI of < 48 hours of age reported that a MAP of < 30mm Hg persisting for over an hour was significantly associated with severe IVH or death within 48 hours.(17) The treatment for cardiovascular insufficiency was based on clinician's discretion and it was not reported how many of the neonates with MAP < 30 mm Hg had signs of poor perfusion, making it difficult to interpret whether MBI was associated with IH or clinical hypotension. On the contrary, LeFlore et al. in their prospective cohort study of VLBW infants of less than 72 hours of postnatal age concluded that the incidence of IVH > grade 2 did not differ between those in whom at least one MAP was less than 30 mmHg and those in whom all MAPs were > 30 mmHg. (18)

Studies on association between MAP variability and short-term outcomes

Bada et al. in their prospective observational study of VPTI reported that those neonates with severe grade IVH had a greater percentage of time with a coefficient of variation of MAP > 13% in the first 48 hours when compared to their matched controls.(19) D'Souza et al. in their prospective observational study of VPTI of ≤ 10 days reported that the coefficient of variation of the MAP values was higher in neonates on the day of IVH, and a similar trend was apparent on the day before. (20) Cunningham et al. also noted that there was a significant association with increased MAP variability and mortality in VLBW neonates in the 1st week of life. (5)

Studies on association between MAP and cerebral perfusion

Meek et al. in their prospective study of VPTI reported that those neonates with lowest cerebral blood flow (CBF) in the first 24 hours of life had a higher risk of severe IVH when compared to those with a higher CBF.(21) Further, those neonates with lowest CBF had significantly higher MAP in the first 24 hours of life when compared to those with higher CBF. A reasonable inference that could be drawn from this finding is that increased MAP could also result in lower CBF in the first 24 hours of life and might be associated with severe IVH. A similar finding of association between higher MAP in the first 24 hours of life and MBI was reported by Gronlund

et al. in their in their prospective observational study of preterm neonates ≤ 36 weeks. (22) Victor et al. in their prospective study of preterm neonates of < 30 weeks within the first 48 hours of life reported that electroencephalogram became abnormal at a MAP < 23 mm Hg, abnormally high cerebral fractional oxygenation was observed at 20 mm Hg, and peripheral blood flow decreased at a MAP range of 23-33 mm Hg. The authors concluded that cerebral perfusion was probably maintained at MAP levels above 23 mm Hg.(23) Similar findings were reported by Binder-Heschl et al. and Deshpande et al. in their prospective cohort studies.(24,25)

Studies on association between clinical hypotension with or without low MAP and short- and long-term outcomes

Carrapato et al. in their retrospective study reported that in VPTI of less than 72 hours, respiratory distress syndrome, sepsis and anemia were significantly associated with clinical hypotension irrespective of BP readings. (26) The authors also reported that PVL was significantly associated with clinical hypotension with or without low MAP, and not with IH. Fanaroff et al. in their retrospective study of ELBW neonates of < 72 hours of age evaluated treatment of hypoperfusion based on assessment of vital signs and the overall clinical status of the neonate with no specific MAP cut-off to define hypotension.(27) The authors concluded that such an approach was significantly associated with delayed motor development after adjusting for baseline sickness. The authors emphasized the importance of establishing normative values for BP in ELBW infants. MAP < 3rd percentile for GA or < 30 mm Hg with one clinical criteria of decreased urine output (<1 ml/kg/hour for >12 hours) in VLBW neonates of < 7 days of life was evaluated by Kim et al., and the authors reported that after correcting for baseline sickness, hypotension with clinical features of hypoperfusion in the first week was associated with composite outcome of mortality or long term NDI (cognitive and language outcomes). (28)

Studies on association between IH and long-term outcomes

Four observational studies evaluated the composite outcome of mortality or long-term NDI.(4,10,29,30) In three of these studies, neonates with IH who were managed with a restrictive approach was compared with those with no hypotension.(4,29,30). Of these studies, 2 studies (21,32) which had evaluated ELGANs of < 24 hours of age had shown that neonates treated with restrictive approach may possibly have similar long-term NDO when compared to those who did not have hypotension. Another retrospective cohort study which had enrolled ELBW neonates of < 72 hours had indicated that long-term NDO was comparable between the restrictive treatment group and those with no hypotension at all.(4) The study by Pellicer et al. reported which had treated neonates with IH with an active approach and compared the long term outcomes with control subjects with no IH found no No differences between groups were found in the rates of abnormal neurologic status, developmental delay, or combined adverse outcome (death or cerebral palsy or severe neurodevelopmental delay) (unadjusted analysis). This was despite the fact that the control group were less sicker compared to the active treatment group.(31) The authors concluded that inotropes should be used with caution in isolated hypotension in the first 24 h of life.

Most of the aforementioned studies indicate that active approach to the treatment of IH in VPTI may not be associated with additional benefits in terms of improved short- and long-term outcomes. Some studies have also indicated possible harm with the active treatment of IH, especially in the first 24 hours of life. Some studies have indicated that increased MAP variability may also result in poorer outcomes. There were a few studies that suggested that restrictive treatment based on the use of multiple clinical criteria of hypoperfusion without taking into account the MAP values may also result in harm. Hence, it is important that restrictive approach to treatment of hypotension be well defined. It is to be noted that there was wide variability between these studies in relation to the GA and postnatal age of the enrolled

neonates, the definition of hypotension and the time span during which these studies were conducted.

References

1. Batton B, Li L, Newman NS, Das A, Watterberg KL, Yoder BA, Faix RG, Laughon MM, Stoll BJ, Van Meurs KP, Carlo WA, Poindexter BB, Bell EF, Sánchez PJ, Ehrenkranz RA, Goldberg RN, Laptook AR, Kennedy KA, Frantz ID 3rd, Shankaran S, et al.; Eunice Kennedy Shriver National Institute of Child Health & Human Development Neonatal Research Network. Use of antihypotensive therapies in extremely preterm infants. Pediatrics. 2013;131(6):e1865-73.

2. Lee J, Rajadurai VS, Tan KW. Blood pressure standards for very low birthweight infants during the first day of life. Arch Dis Child Fetal Neonatal Ed. 1999 ;81(3):F168-70.

3. Kiss JK, Gajda A, Mari J, Nemeth J, Bereczki C. Oscillometric arterial blood pressure in haemodynamically stable neonates in the first 2 weeks of life. Pediatr Nephrol. 2023 ;38(10):3369-3378.

4. Ahn SY, Kim ES, Kim JK, Shin JH, Sung SI, Jung JM, et al. Permissive hypotension in extremely low birth weight infants (≤1000 gm). Yonsei Med J. 2012;53(4):765-71.

5. Cunningham S, Symon AG, Elton RA, Zhu C, McIntosh N. Intra-arterial blood pressure reference ranges, death and morbidity in very low birthweight infants during the first seven days of life. Early Hum Dev. 1999;56(2-3):151-65.

6. Limperopoulos C, Bassan H, Kalish LA, Ringer SA, Eichenwald EC, Walter G,et al. Current definitions of hypotension do not predict abnormal cranial ultrasound findings in preterm infants. Pediatrics. 2007;120(5):966-77.

7. Liu CH, Peng SC, Jin F, Xia SW. 低血压对胎龄<32周早产儿近期预后的影响 [Influence of hypotension on the short-term prognosis of preterm infants with a gestational age of <32 weeks]. Zhongguo Dang Dai Er Ke Za Zhi. 2022;24(11):1195-1201.

8. Martens SE, Rijken M, Stoelhorst GM, van Zwieten PH, Zwinderman AH, Wit JM et al; Leiden Follow-Up Project on Prematurity, The Netherlands. Is hypotension a major risk factor for neurological morbidity at term age in very preterm infants? Early Hum Dev. 2003;75(1-2):79-89.

9. St Peter D, Gandy C, Hoffman SB. Hypotension and Adverse Outcomes in Prematurity: Comparing Definitions. Neonatology. 2017;111(3):228-233.

10. Trounce JQ, Shaw DE, Levene MI, Rutter N. Clinical risk factors and periventricular leucomalacia. Arch Dis Child 1988; 63: 17–22.

11. Weindling AM, Wilkinson AR, Cook J, Calvert SA, Fok TF, Rochefort MJ. Perinatal events which precede periventricular haemorrhage and leukomalacia in the newborn. Br JObstet Gynaecol 1985; 92(12): 1218 – 1223.

12. Batton B, Li L, Newman NS, Das A, Watterberg KL, Yoder BA, Faix RG, et al; Eunice Kennedy Shriver National Institute of Child Health and Human Development Neonatal Research Network. Evolving blood pressure dynamics for extremely preterm infants. J Perinatol. 2014 ;34(4):301-5.

13. Lee, A & Ye, Xiang & Shah, PS.Permissive Approach and Relationship to Outcomes of Infants < 27 Weeks Gestation. Hong Kong Journal of Paediatrics. 2012 (1-6).

14. Liu TX, Shi LP. 超早产儿生后72 h内低血压发生的危险因素及预后分析 [Risk factors and prognosis of hypotension within 72 hours after birth in extremely preterm infants]. Zhongguo Dang Dai Er Ke Za Zhi. 2023 Oct 15;25(10):1001-1007. Chinese.

15. Fernandez E, Watterberg KL, Faix RG, Yoder BA, Walsh MC, Lacy CB et al; Eunice Kennedy Shriver National Institute of Child Health and Human Development Neonatal Research Network. Definitions of cardiovascular insufficiency and relation to outcomes in critically ill newborn infants. Am J Perinatol. 2015 ;32(11):1024-30.

16. Watkins AMC, West CR, Cooke RWI. Blood pressure and cerebral haemorrhage and ischaemia in very low birthweight infants. Early Hum Dev 1989; 19: 103.

17. Miall-Allen VM, de Vries LS, Whitelaw AG. Mean arterial blood pressure and neonatal cerebral lesions. Arch Dis Child. 1987 ;62(10):1068-9.

18. LeFlore JL, Engle WD, Rosenfeld CR. Determinants of blood pressure in very low birth weight neonates: lack of effect of antenatal steroids. Early Hum Dev. 2000 ;59(1):37-50.

19. Bada HS, Korones SB, Perry EH, Arheart KL, Ray JD, Pourcyrous M et al. Mean arterial blood pressure changes in premature infants and those at risk for intraventricular hemorrhage. J Pediatr 1990; 117(4): 607–614.

20. D'Souza SW, Janakova H, Minors D, Suri R, Waterhouse J, Appleton G et al. Blood pressure, heart rate, and skin temperature in preterm infants: associations with periventricular haemorrhage. Arch Dis Child Fetal Neonatal Ed 1995; 72(3): F162 – F167.

21. Meek JH, Tyszczuk L, Elwell CE, Wyatt JS. Low cerebral blood flow is a risk factor for severe intraventricular haemorrhage. Arch Dis Child Fetal Neonatal Ed 1999; 81(1): F15 – F18.

22. Gronlund JU, Korvenranta H, Kero P, Jalonen J, Valimaki IA. Elevated arterial blood pressure is associated with peri-intraventricular haemorrhage. Eur J Pediatr 1994; 153(11): 836–841.

23. Victor S, Marson AG, Appleton RE, Beirne M, Weindling AM. Relationship between blood pressure, cerebral electrical activity, cerebral fractional oxygen extraction, and peripheral blood flow in very low birth weight newborn infants. Pediatr Res. 2006 ;59(2):314-9.

24. Binder-Heschl C, Urlesberger B, Schwaberger B, Koestenberger M, Pichler G. Borderline hypotension: how does it influence cerebral regional tissue oxygenation in preterm infants? J Matern Fetal Neonatal Med. 2016;29(14):2341-6.

25. Deshpande P, Barbosa de Olivera C, Jain A, Hahn C, Shah PS, Guerguerian AM, McNamara PJ. Relationship between cerebral oxygenation, cardiac output, and blood pressure during transitional period in extremely low gestational age neonates. Front Pediatr. 2023;11:1187769.

26. Carrapato MRG, Andrade T, Caldeira T. Hypotension in small preterms: what does it mean? J Matern Fetal Neonatal Med. 2019;32(23):4016-4021.

27. Fanaroff JM, Wilson-Costello DE, Newman NS, Montpetite MM, Fanaroff AA. Treated hypotension is associated with neonatal morbidity and hearing loss in extremely low birth weight infants. Pediatrics. 2006 ;117(4):1131-5.

28. Kim TH, Moon CJ, Sung IK, Youn YA. Hypotension within 1 week of life associated with poor short- and long-term outcomes in very low birth weight infants. Cardiol Young. 2018 ;28(8):1037-1041.

29. Batton B, Zhu X, Fanaroff J, Kirchner HL, Berlin S, Wilson-Costello D, et al. Blood pressure, anti-hypotensive therapy, and neurodevelopment in extremely preterm infants. J Pediatr. 2009;154(3):351-7, 357.e1.

30. Logan JW, O’Shea TM, Allred EN, Laughon MM, Bose CL, Dammann O, et al; ELGAN Study Investigators. Early postnatal hypotension is not associated with indicators of white matter damage or cerebral palsy in extremely low gestational age newborns. J Perinatol. 2011 ;31(8):524-34.

31. Pellicer A, Bravo MC, Madero R, Salas S, Quero J, Cabañas F. Early systemic hypotension and vasopressor support in low birth weight infants: impact on neurodevelopment. Pediatrics. 2009 ;123(5):1369-76.

S5 Table: Data extraction table

BPD_OBS				
**Author**	**RR**	**lower**	**upper**	**Postnatal age**
**Aladangady 2023**	1.35	0.98	1.89	<72 h
**Durrmeyer 2016**	0.94	0.47	1.87	<72 h
**Faust 2015**	2.40	1.82	3.16	<24 h
**Kuint 2008**	1.04	1.02	1.06	<24 h

MBI_NEW_OBS				
**Author**	**RR**	**lower**	**upper**	**Postnatal age**
**Aladangady 2023**	0.58	0.34	1.01	<72 h
**Durrmeyer 2016**	0.31	0.15	0.64	<72 h
**Kuint 2008**	2.61	1	7.12	<24 h
**Dammann 2002**	1.40	0.60	3.20	<24 h
**Faust 2015**	1.86	1.43	2.42	<24 h

Mortality_OBS				
**Author**	**RR**	**lower**	**upper**	**Postnatal age**
**Aladangady 2023**	0.72	0.46	1.14	<72 h
**Durrmeyer 2016**	0.69	0.36	1.31	<72 h
**Faust 2015**	1.48	0.92	2.38	<24 h

NEC stage 2 or more_OBS				
**Author**	**RR**	**lower**	**upper**	**Postnatal age**
**Aladangady 2023**	0.61	0.40	0.93	<72 h
**Durrmeyer 2016**	0.66	0.18	2.38	<72 h

	events in intervention group	total number of patients in intervention group	events in control group	total number of patients in control group	intervention name	control name								Year of study
Permissive hypotension vs GA based RCTs														
Mortality														
Study	Ee	Ne	Ec	Nc	Intervention.group	Control.group	Gestational.age.in	Birth.weight.inclu	settings.low.high		Type of study	BP value used	Invasive or Non-	
Pereira 2018	7	39	2	21	PH	<30 mmHg	<29	NI	high		RCT	<GA <30mmHg	Both	2013-2015
HIP 2021	6	29	7	29	PH	<GA	<28	NA	High		RCT	<GA	IBP	2015-2016
Severe brain injury (severe IVH or >= grade 3 PVL) (RCTs)														
Author	Ee	Ne	Ec	Nc	Intervention.group	Control.group	Gestational.age.in	Birth.weight.inclu	settings.low.high		Type of study			
Pereira 2018	0	39	0	21	PH	<30 mmHg	<29	NI	high		RCT	<GA <30mmHg	Both	2013-2015
HIP 2021	5	29	5	29	PH	<GA	<28	NI	high		RCT	<GA	IBP	2015-2016
NEC >=Stage 2 (RCT)														
Author	Ee	Ne	Ec	Nc	Intervention.group	Control.group	Gestational.age.in	Birth.weight.inclu	settings.low.high					
Pereira 2018	9	39	6	21	PH	<30 mmHg	<29	NI	high		RCT	<GA <30mmHg	Both	2013-2015
HIP 2021	1	29	4	29	PH	<GA	<28	NI	high		RCT	<GA	IBP	2015-2016
PDA														
Author	Ee	Ne	Ec	Nc	Intervention.group	Control.group	Gestational.age.in	Birth.weight.inclu	settings.low.high					
Pereira 2018	16	39	8	21	PH	<30 mmHg	<29	NI	high		RCT	<GA <30mmHg	Both	2013-2015
HIP 2021	1	29	0	29	PH	<GA	<28	NI	high		RCT	<GA	IBP	2015-2016
Moderate to severe BPD														
Author	Ee	Ne	Ec	Nc	Intervention.group	Control.group	Gestational.age.in	Birth.weight.inclu	settings.low.high					
Pereira 2018	26	39	12	21	PH	<30 mmHg	<29	NI	high		RCT	<GA <30mmHg	Both	2013-2015
HIP 2021	17	29	14	29	PH	<GA	<28	NI	high		RCT	<GA	IBP	2015-2016

a.Author names who did the data extraction:Vadakkencherry Viraraghavan Ramaswamy, Gunjana Kumar

b.Dates of data extraction: First phase, 17th July 2023 - 1st August 2023; Second phase for updated search: 3rd April 2024 to 7th April 2024

c. All the data utilized in the meta-analyses for the various outcomes are provided

d. All included studies satisfy the eligibility criteria of our *a priori* planned inclusion criteria specified in the registered protocol

e. This systematic review and meta-analyses included only published data and no additional data was obtained from either the primary researchers or other sources
