# Supplementary material for: Active versus restrictive approach to isolated hypotension in preterm neonates: A Systematic Review, Meta-analysis and GRADE based Clinical Practice Guideline
Source: PLoS One. 2025 Mar 18;20(3):e0309520. doi: 10.1371/journal.pone.0309520 (PMC11918419; doi:10.1371/journal.pone.0309520)
Supplement: S2 File — S1 Table. Characteristics of the studies included in the meta-analysis. S2 Table. Characteristics of the studies included in the narrative review. (DOCX) [file pone.0309520.s002.docx]

Table S1. Characteristics of the studies included in the meta-analysis.

| **Randomized controlled trials (RCTs)** | | | | | | | |
| --- | --- | --- | --- | --- | --- | --- | --- |
| Author  Country  Study design | GA (w)  Mean ±  SD  or Median (IQR)  or  GA cut-off for enrolment | BW (g)  Mean ±  SD  or Median (IQR) | Active Intervention Group * | Restrictive treatment group * | Other comments | | |
| Dempsey 2021  Europe, Canada  RCT  HIP trial  Multi-centre | **I:** 25.4 ± 1.3  **C:** 25.3 ± 1.5 | **I:** 745 ± 171  **C:** 683 ± 146 | **I:** Normal saline bolus with dopamine infusion (n = 29) | **C:** Normal saline bolus with placebo infusion (n = 29) | • Definition of hypotension: MAP < GA for ≥  15 min without any signs of poor perfusion  • IBP was used  • GA cut-off: <  28 w  • Postnatal age of enrolment: < 72 h  • Trial was terminated early due to enrolment issues (only 7.7% of the planned sample size were recruited)  • Primary outcome of survival until 36 w of postmenstural age free of severe brain injury was comparable between the two groups  • Additional treatments for low BP were used less frequently in the active arm  • The study found that the incidence of low BP was less than what was published in literature and approximately less than 25% of neonates had at least one episode of hypotension as defined by the authors. The mean time to presentation with low BP was within 6 h  • The authors concluded that performing studies of cardiovascular instability in extreme preterm infants is challenging, and that alternative trial designs/ consent strategies need to be explored | | |
| Pereira 2019  USA  3 armed pilot RCT | **I:** 25.7  (23.4–28.9)  **C1:** 25.8  (23.3–28.7)  **C2**: 25.6  (23.7–28.7) | **I:** 760  (580–1000)  **C1:** 790  (540–1470)  **C2:** 810  (470–1180) | **Active arm (I1):** MAP <  30 mmHg for more than 15 consecutive min (n = 19)  **Moderate arm (I2):** MAP < GA for more than 15 consecutive min (n = 20) | **Permissive arm (C1):** Signs of poor perfusion or MAP <  19 mm Hg (n = 21) | • Both IBP and NIBP were used  • GA cut-off: <  29 w  • Postnatal age of enrolment: < 12 h  • There were no differences in hemodynamic or EEG variables.  • There were no significant differences in clinical outcomes, including mortality, duration of care, respiratory or gastrointestinal complications, ROP or renal variables.  • The authors concluded that since the trial was underpowered, future RCTs which are adequately powered are warranted | | |
| **Non-RCTs** | | | | | | | |
| Aladangady 2023  U.K.  Retrospective cohort study | **I:** 26.2 (26.0–26.3)  **C:** 26.2  (25.9–26.4) | **I:** 834.2 (814.3–854.2)  **C:** 853.1 (824.9–881.2) | **I:** Aimed to maintain MAP  >30 mm Hg without any clinical signs of hypoperfusion (n = 263) | **C:** Inotropes initiated only when neonate had hypotension associated with signs of poor perfusion developed signs of hypotension (CFT > 3 sec, urine output < 1ml/kg/h, lactate > 3mmol/L, base deficit > 8mmol/l and/or increasing oxygen requirement) (n = 408) | • Definition of hypotension: MAP <  30 mm Hg  • NIBP or IBP not mentioned  • GA cut-off: <  29 w within 72 h of life  • The mean GA, BW, admission temperature, clinical risk index for babies score and first haemoglobin of infants were comparable between the groups  • After adjustment for baseline sickness, **the risk of mortality [aOR: 1.38 (0.88–2.16)] and IVH>  grade 2 [aOR: 1.71 (0.99–2.97)] was similar in both the groups. The risk of NEC ≥  stage 2 was higher in the permissive group [aOR: 1.65 (1.07–2.50)].** There were no differences in the risk of PDA requiring medical or surgical management or BPD between the two groups | | |
| Batton 2016  U.S.A.  Prospective cohort study  Multi-centre  NICHD NRN | 23–26 ^6/7^ w | < 24 h | **I:** Neonates in whom the BP did not rise as expected in the first 24 h of life and were treated with volume expansion and/ or inotropes (n = 198) | **C:** Neonates who were not treated irrespective of whether the BP rose as expected or not (n = 158) | • Definition of hypotension: There was no specific cut-off for defining hypotension. The criteria used to treat was based the expected rise of MAP which was ≥  5 mm Hg from 4–24 h  • NIBP and IBP were utilized  • Multivariate logistic regression adjusting for confounders indicated that any anti-hypotensive therapy was associated with increased odd of risk or NDI at 18- 22 months’ CA **[aOR: 1.84 (1.10–3.10)]**  • **The authors concluded that in this this sub-group of neonates, antihypotensive therapy in the first 24 h of life was associated with increased risk of death or NDI at 18**–**22 months’ CA which cannot be explained by the differences in baseline sickness of the different groups** | | |
| Dammann 2002  U.S.A  Case-control study | Mean: NA  Inclusion criterion: ≤  28 w | NA | Case control study of sub-group of ELGANs with cranial echolucencies (IVH>  grade 2, cystic PVL or ischemic lesions). 3 epochs were compared based on postnatal age: epoch 1 ( < 24 h, n = 243), epoch 2 (24 h - 4 d, n = 236) and epoch 3 (≥4 d -7 d, n = 205). | NA | • Definition of hypotension: MAP in the lowest quartile for the neonate’s week of GA. --Hypotensive neonates were treated with vasopressors or volume expanders. Not mentioned if clinical evidence of hypoperfusion was considered  • Mode of BP measurement: NI  • Regression analysis after adjusting for baseline sickness (including use of volume expansion and inotropes) indicated that hypotension was not associated with cranial echolucencies: **epoch 1:** **[aOR: 1.40 (0.60–3.20)], epoch 2: [aOR: 1.50 (0.50–3.90)], epoch 3: [aOR: 1.00 (0.40–2.70)] - Authors concluded that systemic hypotension in the first week might not be associated with major brain injury and cautioned against the use of volume expansion or inotropes to treat isolated hypotension** | | |
| Durrmeyer 2017  France  Prospective Observational Study  Multi-centre  EPIPAGE study | 24 - 28 w within 72 h of life | NA | **I:** minMAP < GA with treatment (n = 119) | **C:** minMAP < GA without treatment (n = 119) | • Modality of BP measurement not mentioned  • Propensity matched cohort analysis was performed  • **Treated infants had a significantly higher survival rate without major morbidity and lower rate of severe cerebral abnormalities**  • **The authors reported that untreated isolated hypotension (without any signs of poor perfusion) was associated with poor short-term outcomes. There was a trend towards increased odds of survival without major morbidities defined as NEC** ≥  **stage 2, severe cerebral abnormalities, severe BPD or severe ROP was higher in the untreated group [aOR: 1.71 (1.00–2.97)]. In the sensitivity analysis, the sub-group of neonates with more severe isolated hypotension (minMAP** ≤  **GA-5), the aOR was 3.15; 95% CI 1.28 to 7.74 indicating stronger association**  • **The authors concluded that not treating early isolated hypotension should be cautiously considered in extremely premature infants.** | | |
| Faust 2015  Germany  Retrospective cohort  Multi-centre  German Neonatal Network | **I:** 28.10 ± 2.2  **C:** 28.02 ± 2.3 | **I:** 1015 ± 305  **C:** 1013 ± 295 | Outcomes of neonates with minMAP lower than median minMAP of all neonates of the corresponding gestational age in completed weeks was studied | NA | • Definition of hypotension: Isolated MAP as mentioned in the columns before. In neonates with a GA of ≤  29 weeks’, the median minMAP was 1–2 mm Hg lower than GA in completed weeks. BP in the lowest quartile of minMAP was at least 5 mm Hg lower than gestational age in completed weeks. minMAP lower than median minMAP of all patients of the corresponding gestational age in completed weeks was also compared post-hoc - NIBP or IBP were utilized - - GA cut-off: <  32 w within the first 24 h of life **- In a multivariate regression model, minMAP was significantly associated with increased odds of any IVH (aOR 0.97/mm Hg, 95% CI 0.95 to 0.99, p = 0.006), BPD (aOR 0.96/mm Hg, 95% CI 0.94 to 0.98, p < 0.001) and death (aOR 0.95/mm Hg, 95% CI 0.90 to 0.99, p = 0.026) in neonates who were not treated with vasoactive drugs on the first day of life.** **- Further, after adjusting for the confounders, the risk of any IVH was higher in the neonates who were not treated and had minMAP in the lowest quartile for gestational age when compared with infants with minMAP ≥ 25^th^ percentile (IVH 18.4% vs 14.3%, p = 0.004)** **- However, multivariate logistic regression indicated that treatment with inotropes in the first 24 h for the whole cohort who had isolated hypotension was associated with increased risk of any IVH [aOR: 1.86 (1.43–2.42)], BPD (need of O2 support or CPAP at 36 weeks’ PMA) [aOR: 2.40(1.82– 3.16)], and a trend towards with increased mortality [aOR: 1.48 (0.92–2.28)] - From the above results, it is unclear whether isolated hypotension as defined by the authors should be treated or not as one adjusted analysis showed treatment of isolated hypotension is associated with poor outcomes, whilst the second multi-variate regression analysis controlling for treatment of hypotension showed poor outcomes in untreated neonates with isolated hypotension** | | |
|  |  |  |  |  | Lowest MAP during the first 24 h and gestational age (completed weeks) | | |
|  |  |  |  |  | GA | n | min MAP [Median (IQR)] |
|  |  |  |  |  | 22 | 25 | **21** (18–25) |
|  |  |  |  |  | 23 | 178 | **21** (19–24) |
|  |  |  |  |  | 24 | 339 | **22** (20–25) |
|  |  |  |  |  | 25 | 431 | **24** (21–26) |
|  |  |  |  |  | 26 | 583 | **24** (21–28) |
|  |  |  |  |  | 27 | 666 | **26** (22–29) |
|  |  |  |  |  | 28 | 725 | **27** (24–31) |
|  |  |  |  |  | 29 | 725 | **29** (25–32) |
|  |  |  |  |  | 30 | 709 | **30** (27–34) |
|  |  |  |  |  | 31 | 526 | **31** (27–35) |
|  |  |  |  |  | All | 4907 | **27** (23–31) |
| Gogcu 2020  U.S.A.  Case-control study | **I:** 25 (23–28)  **C:** 26 (23–30) | **I:** 780 (512–955)  **C:**844 (635–969) | Case control study of neonates with sensineural hearing loss detected at 12–24 months’ and their exposure to inotropes in the first 24 hours was evaluated (n = 25) | NA | • Definition of hypotension: MAP < GA irrespective of clinical symptoms or signs  • IBP was used  • ELBW neonates <  24 h of postnatal age were enrolled - Multi-variate analysis showed that treatment of hypotension without any clinical signs or symptoms in the first 24 h of life was associated with increased risk of hearing loss **[aOR: 3.60; 95% CI: (1.30–9.70)]** when adjusted for gestational age and maternal treatment with antenatal steroids | | |
| Kuint 2008  Israel  Case-control study | 27.9 ± 81.90 | 1004 ± 233 | Isolated hypotension defined as MAP < GA was treated (n = 109). This was a matched case-control study, only the adjusted analysis is reported | NA | • Definition of hypotension: Isolated hypotension defined as MAP < GA was treated  • NIBP or IBP was used  • VLBW neonates of <  24 h were enrolled  • Multivariate logistic regression revealed that treatment of isolated hypotension was associated **with an increased risk of PVL [aOR: 2.61 (95% CI 1.0– 7.12)], severe ROP [aOR: 1.04 (95% CI 1.02– 1.06)], BPD [aOR: 1.04 (95% CI 1.02– 1.06)] and NDI at 2 years of age [aOR: 5.40 (95% CI 1.29– 22.7)]**  • Further, **lowest MAP [24.1 ±  3.2 mm Hg] was an important predictor of IVH>  grade 2 [OR: 4.58 (95% CI 1.45– 14.50)]**  • The authors concluded that treatment of isolated hypotension in the first 24 h is associated with poor short and long term outcomes. | | |

GA: Gestational age, BW: Birth weight, w: Weeks, g: Grams, I: Intervention group, C: Control group, SD: Standard deviation, IQR: Interquartile range, RCT: Randomized controlled trial, h: hours, d: day, BP: Blood Pressure, IVH: Intraventricular hemorrhage, BPD: Bronchopulmonary dysplasia, NEC: Necrotising enterocolitis, ROP: Retinopathy of prematurity, PDA: Patent ductus arteriosus, PVL: Periventricular leukomalacia, MAP: Mean arterial pressure, CFT: Capillary refilling time, IBP: Invasive BP, NIBP: Non-invasive BP, ELGANs: Extremely low gestational age neonates, ELBW: Extremely low birth weight, VLBW: Very low birth weight, EEG: Electroencephalogram, aOR: Adjusted odds ratio, 95% CI: 95% confidence interval, NDI: Neurodevelopmental impairment, CA: Corrected age, NA: Not applicable, NI: No information

*Active intervention group: Treatment with volume expansion and/ or inotropes for Isolated hypotension. Isolated hypotension was defined as low mean arterial blood pressure (MAP) as ascertained by the investigator which could be based on definitions such as MAP less than gestational age, MAP value below the 5^th^ centile for the particular gestational age, MAP less than 30 mm Hg without any clinical or biochemical evidence of hypoperfusion.

*Restrictive treatment group:

a. Treatment with volume expansion and/ or inotropes in preterm neonates with hypotension only when clinical and/ or biochemical features of poor perfusion were present. The clinical and/ or biochemical signs of hypoperfusion were defined as follows: Tachycardia (>160–170 beats/min), prolonged capillary refilling time ( > 3–4 seconds), low peripheral pulses, decreased urine output ( < 1 ml/kg/ hour for 4 -6 hours, physiological oliguria or anuria should also be considered), increasing lactate levels ( > 3–4 mmol/L) and base deficit ( > 8 meq/L).

b. No treatment of isolated hypotension.

Table S2. Characteristics of the studies included in the narrative review.

| **Author**  **Country**  **Study design** | **GA (w)**  Mean ±  SD or  Median (IQR)  or  **GA cut-off for enrolment** | **BW (g)**  Mean ±  SD or  Median (IQR) | **Postnatal age till MAP was monitored** | **Intervention/ Control ***  **(n)** | **Other comments** |
| --- | --- | --- | --- | --- | --- |
| Ahn 2011  Korea  Retrospective study | I: 25.6 ± 2.1  C: 25 ± 2.2 | I: 764 ± 147  C: 672 ± 152 | < 72 h | - Treatment (T) group: MAP < GA  and received intervention including volume pushes, vasopressors or corticosteroids (n = 47)  - Permissive Group (P) group: MAP < GA with signs of good perfusion  and no intervention (n = 104) | • Definition of hypotension: MAP < GA  • NIBP and IBP were used  • ELBW neonates were enrolled  • Three groups were compared: Normotensive group (N) - MAP>  GA, 2) Permissive hypotension group (P) - MAP < GA with signs of good perfusion and no intervention, and 3) Treated hypotension group (T) - MAP < GA and treated with volume expansion/ vasopressors/ corticosteroids  • BW and Apgar scores were significantly lower in the T group, but proportion of neonates with pathologically confirmed chorioamnionitis was higher in P group  • However, after adjusting for baseline sickness, **mortality [aOR: 3.30 (1.30–8.50)], IVH>  stage 2 [aOR: 7.40 (2.60–21.50)] and BPD (O_2_ requirement at 36 weeks’ PMA) [aOR: 3.60 (1.30–10.30)] were significantly higher in the T group** when compared to **N group**. NEC ≥  stage 2, ROP requiring treatment, PVL were comparable between the P and N groups. On the contrary, **the P group had comparable outcomes to N group. No comparison adjusted for baseline sickness was calculated for T vs. P group**  • Long term neurodevelopmental outcomes namely, PDI <  75, MDI <  75, hearing abnormalities, cerebral palsy were not significantly different between the N and P groups (Unadjusted for baseline sickness). Due to substantial loss of follow up in T group, long term neurodevelopmental outcomes were not assessed.  • **The authors concluded that close follow up of ELBW neonates with isolated hypotension with no signs/ symptoms of poor perfusion, and without intervention could prevent unnecessary use of inotropes and may result in neurological outcomes similar to neonates with normotension** |
| Bada 1990 U.S.A Prospective observational study | 29 ±  2.4 | 1158 ±  235 | < 48 h | The study aimed to measure the BP values in the first 48 h and evaluate the association of MAP with PVH (n = 100) | • No specific definition for hypotension was mentioned. No information was provided on management of hypotension  • IBP was used  • **Those neonates with PVH had a greater percentage of time with a coefficient of variation of MAP >  13% or < 3%, when compared to their matched control subjects (p < 0.005)**  • **In the sub-group of ELBW neonates (n = 28) who developed PVH, the MAP was observed to be consistently low in the first 48 h of postnatal life** |
| Batton 2013  U.S.A  Multi-centre, NRN, NICHD  Prospective cohort | I: 25.1 ± 1.1  C: 25.5 ± 0.9 | I: 698 156  C: 764 ± 161 | < 24 h | - Preterm neonates who received anti-hypotensive therapy vs. no therapy were compared (n = 367)  - Of the 367 neonates enrolled, 307 received anti-hypotensive therapy or a vasoactive drug | • Definition of low BP: 15 definitions were used: 1,2 ≥  3 systolic, diastolic or mean value ≤  5th centile. ≥  3 MAP values less than or equal to GA, ≥  3 values ≤  25 mm Hg (at least 1 h apart). These values were not necessarily consecutive.  • IBP and NIBP were used  • GA cut-off: <  27 w  • **Authors noted that anti-hypotensive therapy usage was lowest in neonates who did not have MAP ≤  25 mm Hg or GA equivalent (28%). Those** with ≥  **3 BP values** ≤  **5^th^ percentile of systolic BP (83%), diastolic BP (93%) or MAP (97%) was associated with highest anti-hypotensive therapy rates, and most of them had multiple low BP values.**  • **Anti-hypotensive therapy was also initiated in neonates without low BP and in some neonates with low BP, it was prescribed and that additional factors apart from low BP contributed to provide anti-hypotensive therapy**  • **Regression analysis controlling for severity of illness indicated that, there were no significant differences between groups in survival (through first postnatal week or until discharge) or in-hospital morbidity rates (IVH ≥  grade 2, severe ROP, survival until discharge without morbidities NEC, ROP, IVH>  grade 2 or cystic PVL) (aOR not provided)**  • The authors concluded that factors other than BP also contributed to the decision to use antihypotensive therapies. **The neonatal outcomes did not improve with antihypotensive therapy for any of the 15 definitions and that a numeric cut-off for initiating anti-hypotensive therapy may not be utilized as outcomes were similar or worser in the treated group vs. the not treated group** |
| Batton 2009  U.S.A  Retrospective  Study | G1: 24.7 ±  0.6  G2: 24.6 ±  0.7  G3: 24.4 ±  0.6 | G1: 707 ±  105  G2: 698 ±  116  G3: 674 ±  116 | < 24 h | The objective of the study was to compare the neurodevelopmental outcomes of 3 cohorts: untreated with normal BP (G1, n = 67), untreated with low BP (G2, n = 31) and treated with low BP and clinical signs of low perfusion (G3, n = 70). The G3 was treated if there were clinical and/ or biochemical evidence of poor perfusion along with low BP | • Low BP was defined as ≥  3 MAP readings (at least I h apart) ≤  25 mm Hg in the first 72 hours  • NIBP and IBP were utilized  • GA cut-off: <  26 w  • A total of 75% survived to be discharged from the hospital, and 95% of survivors had ND assessment  • **After adjusting for potential confounders (receipt of antenatal corticosteroids, gender, maternal education, postnatal dexamethasone exposure and BPD), multi-variate logistic regression indicated that infants with normal BP were less likely than infants with treated or untreated low BP to have an MDI <  70, any NDI at 18 to 22 months PMA, odds of the combined outcome of death or MDI <  70 and death or any NDI. Infants with low BP had poor outcomes regardless of treatment status**  • **When G3 vs. G2 were compared, there were no difference in the outcomes of any NDI [aOR: 1.50 (0.50–4.80)] and any NDI or mortality [aOR: 1.70 (0.60–5.00)]**  • The authors concluded that neonates with **low BP (≤25 mm Hg)** regardless of whether they are treated or not (G2 and G3) have poorer neurodevelopmental outcomes and that early low BP may be associated with a poorer outcomes.  • **The G3 vs. G2 comparison showing no difference in outcomes indicate that treatment of isolated hypotension may not be beneficial** |
| Batton 2007  U.S.A  Retrospective study  Multi-centre  NICHD NRN | G1: 24.1 ± 0.8  G2: 24.3 ± 0.7 | G1: 694 ± 104  G2: 741 ± 133 | < 7 d | The objective of this study was to construct a nomogram of SBP, DBP and MAP for neonates of 23–25 w GA. The authors compared the outcomes of those neonates with at least 2 MAP readings 1 hour apart of ≤  25 mm Hg with no signs of poor perfusion (G1, low BP group, n = 38) with that of neonates who were deemed normotensive (G2, high BP group, n = 48) | • NIBP or IBP were used  • For the 86 infants who were not treated for hypotension, MAP increased by 0.3 mm Hg/ h in the first 24 h, 0.1 mm Hg/h from the 25^th^ to the 48^th^ h, and then stabilized for the remainder of the first w  • **G1 and G2 did not differ in their baseline characteristics. There were no differences in the incidence of IVH, PVL, NEC, ROP, or BPD between the two groups**  • **A third group of neonates who were sicker and treated for signs of poor perfusion was also compared. These neonates had poorer outcomes. There was no specific BP cut off at which these neonates were treated.**  • **Since the outcomes were comparable for G1 vs G2, it could be inferred that isolated hypotension (at least 2 readings of MAP ≤  25 mm Hg) without any clinical symptoms in the first 7 d may not be treated** |
| Batton 2014  U.S.A  Prospective Observational study  Multi-centre  NICHD NRN | 23–26 w | NA | < 24 h | The authors studied the changes in BP in neonates in the first 24 h of life (n = 364). Two cohorts of preterm neonates: those with who were treated for hypotension (n = 164) and those who were not (n = 430) was evaluated. Whether the treated neonates had poor signs/ symptoms of poor perfusion was not recorded | • There was no standard definition for hypotension  • NIBP and IBP were used  • GA cut-off: <  27 w  • BP decreased in the first 3 h, reached a nadir at 4–5 h and then increased at a rate of 0.2 mm Hg/ h. The rise in BP from hour 4–24 h was similar for untreated infants and untreated neonates.  • BP trended to be lower as GA decreased, but there was wide variation at each GA  • **The authors concluded that BP increases spontaneously over the first 24 h in this sub-group of preterm neonates and that the rate of rise in BP was similar between treated vs. untreated neonates** |
| Batton 2012  U.S.A  Pilot RCT | < 27 w | NI | < 24 h | 2 x 2 factorial design  Dopamine/ Hydrocortisone vs. placebo for neonates with isolated hypotension | Definition of hypotension:   \| Postnatal (h) \| 1–6 \| 7–12 \| 13–18 \| 19–24 \| \| --- \| --- \| --- \| --- \| --- \| \| MAP mm Hg \| 24 \| 25 \| 26 \| 27 \|   • IBP was used  • Up to 20 ml/kg of volume expansion was permissible before study entry  • Only 10 infants were enrolled, the RCT was not feasible  • The authors concluded that the RCT was not feasible because of less number of neonates being eligible and issues with consent. Further, the authors were of the view that such an RCT might require a waiver of consent as they consider the PICO format to be research in emergency care |
| Binder-Heshchl 2017  Austria  Prospective observational study | I:33.4  ± 1.9  C: 33.3  ±  1.3 | I: 2016.5  ±  548.5  C: 1924.7  ±  451.9 | <24 h | -The intervention group (n = 17) had isolated hypotension. The control group had no hypotension (n = 29).  -The cerebral regional tissue oxygenation (crSO_2_), peripheral oxygen saturation (SpO_2_), heart rate (HR) and MAP were monitored for the first 24 h, starting within the first 6 h after birth.  -The cerebral fractional tissue oxygen extraction (cFTOE) was also calculated. Preterm neonates with and without isolated hypotension arterial hypotension were compared to each other. | • IBP was monitored  • Definition of low BP was MAP < GA  • All the parameters were similar in either of the two groups and was not statistically significant  • The authors concluded that cerebral autoregulation is maintained in case of isolated hypotension in the first 24 h of life. |
| Carrapato 2018  Portugal  Retrospective cohort | 28.5 ± 2.1 | 1182 ± 379 | < 72 h | Neonates with isolated hypotension, hypotension with no clinical or biochemical features of poor perfusion, normotension with clinical or biochemical features of poor perfusion were studied (n = 164) | • Definition of low BP was MAP < GA  • GA cut-off: <  32 w  • 29.9% had MAP < GA; 14% had low BP but no clinical manifestation of poor perfusion; 19.5% had no hypotension but had clinical signs of hypoperfusion. Only 15.9% had hypotension along with signs of hypoperfusion.  • **RDS, sepsis, anemia were significantly associated with clinical hypotension irrespective of BP readings. IVH and PDA had significant association with GA and BW, independent of isolated hypotension or clinical hypotension.**  • **PVL was significantly associated with clinical hypotension with or without low MAP, and not with low MAP alone**  • **Authors concluded that if persistent hypotension is to be treated, rather than treating low MAP in isolation, clinical signs of hypotension should guide treatment. Permissive hypotension especially if transient, in the absence of clinical signs of hypoperfusion may be considered** |
| Cunningham 1999  U.K.  Retrospective cohort | 4 groups:  26.0 ± 1.80  27.0 ± 1.70  28.0 ± 1.90  29.0 ± 1.20 | 4 groups:  645 ± 81  876 ± 73  1118 ± 86  1380 ± 76 | ≤ 7 d | Stable neonates who were not on vasopressor therapy (n = 232) | • The aim of the study was to construct percentile charts of MAP in VLBW neonates, grouped into 4 categories based on gestational age: ≤  750 g, 751–1000g, 1001–1250 g, 1251–1500 g  • IBP was used. The authors reported a progressive increase in MAP in the first 7 days of life  • **There was no significant association between the percentage of time the neonates’ MAP was less than GA after adjusting for BW, GA and IVH. There was a significant association with increased MAP variability and mortality in the first 7 days of life**  • **There was a significant association between isolated low MAP (d1, d3) (p < 0.05), variable MAP (d1-d7) (p < 0.01) and maximum MAP (d2) (P < 0.05); and IVH (unadjusted association). No neonate with IVH>  grade 2 had more than 10% of MAP observations less than their gestational age**  • **No association with MAP was found for the other outcomes of PVL or ROP** |
| Dempsey 2009  Canada  Retrospective study | G1: 26.6 ± 1.60  G2: 26.6 ± 1.60  G3: 25.2 ± 1.62 | G1: 828 ± 144  G2: 742 ± 131  G3: 728 ± 149 | < 72 h | Three groups of neonates were evaluated.  G1 (n = 52): BP never less than GA  G2 (n = 34): BP < GA but signs of good perfusion, not treated: permissive hypotension group  G3 (n = 18): BP < GA with signs of poor perfusion and treated | • MAP in the permissive group increased from 26 mm Hg at 6 h to 31 mm Hg at 24 h  • ELBW neonates were enrolled  • **The authors concluded that G2 had similar outcomes when compared to G1 (unadjusted)**  • **Also, multivariate logistic regression after adjusting for baseline sickness indicated that G3 was associated with poorer composite outcome of mortality or severe IVH, cystic PVL, surgical NEC or gastrointestinal perforation [aOR: 9.70 (2.6–36.00)]**  • **The authors concluded that BP spontaneously improves in ELBW infants during the first 24 h and isolated hypotension may not be treated** |
| Deshpande 2023  Canada  Retrospective study | < 28 | – | <72 h | Cerebral saturation (CrSO2) monitoring and serial echocardiography monitoring in the first 72 h of birth were performed (n = 50). Correlative analyses of CrSO2 and systolic BP, diastolic BP, and MAP were conducted. | • The definition and management of hypotension were subjective and was based on clinicians’ discretion.  • There was only a weak correlation between measures of cardiac output, BP, and middle cerebral artery mean velocities with both CrSO2 and CFTOE in ELGANs during transition.  • The authors concluded that these findings possibly suggest cerebral autoregulation might be maintained in ELGANs in the first 72 h and that prospective studies are warranted to validate their findings. |
| D’Souza 1995  U.K.  Prospective cohort study | ≤ 33 | NI | < 10 d | The objective of the study was to evaluate the association of BP changes with PVH (n = 34) | • Clinical hypotension with poor perfusion was treated. No MAP or BP cut-off for initiation of hypotension was mentioned  • IBP was used  •**The authors reported that the coefficient of variation of the MAP values tended to be higher in infants on the day of PVH, and a similar trend was apparent on the day before.** |
| Fanaroff 2006  U.S.A  Retrospective cohort | I: 24.9 ± 1.7  C: 26.1 ± 1.9 | I: 714 ± 154  C: 768 ± 141 | < 72 h | Comparison of outcomes treated hypotension cohort vs. not treated for hypotension cohort | • Definition of hypotension: Clinicians treated low blood pressure on the basis of associated vital signs, perfusion, and the overall clinical status of the patient. No specific cut-off of MAP to define hypotension  • ELBW neonates were included  • NIBP or IBP were utilized  • **Authors reported that treated hypotension cohort was significantly associated with delayed motor development, defined as a mental developmental index of <  70 after adjusting for baseline sickness**  • **The authors emphasized the importance of establishing normative values for BP in ELBW infants** |
| Fernandez 2015  U.S.A  Retrospective cohort study  Multi-centre  NICHD NRN | Two sub-groups: ≥  37 w and <  37 w were studies | NI | < 72 h | 4 definitions used to treat hypotension were evaluated and their association with short- and long term outcomes were studied:  D1.: 2 consecutive MAP < GA  D2: D1 +  at least one clinical/ bichemical sign of hypoperfusion [poor CFT ( > 3 seconds), oliguria (urine output <  1ml/kg/hour over 6 hours) or serum bicarbonate <  18 and/ or base deficit > 5)]  D3: Receipt of fluid boluses, inotropes or glucocorticoids  D4: Receipt of inotropes | • Overall, multivariable regression analysis indicated that when compared to neonates with no cardiovascular insufficiency, **D1 was not significantly associated with odds of death.** **However D2, D3 and D4 was significantly associated with odds of death, with the association being stronger in the ascending order**  • **D2 included only one clinical sign, of which serum bicarbonate <  18 and base deficit of >  5 might be considered as liberal, especially in preterm neonates**  • The authors had concluded that using a threshold BP alone might not improve clinical outcomes in term or preterm neonates and that they did not explore centile based definition for defining hypotension |
| Gronlund 1994  Finland  Prospective observational study | 32 (26–36) | 1700 (1020–3720) | < 24 h | The authors studied the association of systolic BP, diastolic BP and MAP with major brain injury (n = 42) | • No information regarding the definition of hypotension, treatment criteria for hypotension were provided  • IBP was recorded  • The authors noted that elevated diastolic, mean and systolic BP were significantly associated with major brain injury in preterm neonates |
| Kim 2018  Republic of Korea  Retrospective cohort | 27.37 ±  2.29 | 1061.81 ±  267.73 | < 7 d | Though there were two groups of VLBW neonates, one group who were hypotensive and the other being normotensive, the baseline characteristics indicated that the hypotensive group were sicker (n = 84) | • Definition of hypotension: MAP <  3^rd^ percentile for GA or <  30 mmHg with decreased urine output (<1 ml/kg/hour) for > 12 hours  • IBP was used  • VLBW neonates were enrolled  • **In the multi-variate analysis correcting for baseline sickness, hypotension in the first week, PVL and longer duration of mechanical ventilation was associated with poor long term outcome of mortality or NDI [aOR: 3.53 (1.36–9.30)]**  • **It is to be noted that though the authors report an association of hypotension with poorer long term outcome, the clinical criterion used to initiate treatment was decreased urine output for >  12 hours. Oliguria for such a prolonged period of time in neonates of <  7 d may indicate a longer duration of hypoperfusion and hence delayed treatment of clinical hypotension** |
| Kiss 2022  Hungary  Retrospective cohort study | Both preterm and term neonates were included. The following sub-groups were studied: 25–28 w, 31 w, 34 w, 36 w and 40–42 w. | – | <14 d | - The authors constructed nomograms for the different GA groups.  - Sick neonates requiring mechanical ventilation and inotropes were excluded.  - 25–28 w: n = 27; 29–32 w: n = 150; 33–36 w: n = 201. | • NIBP was measured  • The authors reported that the average rates of change of the systolic BP in the first five days for the sub-groups 25–28 w, 32 w and 36 w were: 3.11 mmHg/day, 2.49 mmHg/day and 1.88 mmHg/day, respectively. The corresponding MAP values were 2.61 mmHg/day, 2.06 mmHg/day and 1.51 mmHg/day, respectively. The corresponding diastolic BP values were 2.30 mmHg/day, 1.79 mmHg/day and 1.28 mmHg/day, respectively.  • **The systolic BP, diastolic BP and MAP rise were steeper in the preterm group than in the term group in the first 3 days of life indicating that there is no specific BP cut-off to define hypotension based on BP values alone in the first 3 days of life.**  • This study showed how BP varies with gestational age and birth weight. |
| Lee 1999  Singapore  Prospective cohort study | 28.00 ±  2.80 | 996 ±  281 | < 24 h | - A nomogram for non-sick babies not on inotropes was derived from 61 VLBW neonates (ELBW: n = 28; ≤  28 w =  34) | • IBP was used.  • The lower 95% CI of MAPs were as follows:   \| **GA** \| 23 \| 24 \| 25 \| 26 \| 27 \| 28 \| 29 \| 30 \| 31 \| 32 \| 33 \| 34 \| 35 \| \| --- \| --- \| --- \| --- \| --- \| --- \| --- \| --- \| --- \| --- \| --- \| --- \| --- \| --- \| \| **1^st^ 12h** \| 20 \| 21 \| 22 \| 23 \| 25 \| 26 \| 27 \| 28 \| 29 \| 30 \| 31 \| 32 \| 33 \| \| **12-24h** \| 20 \| 22 \| 23 \| 25 \| 27 \| 28 \| 29 \| 30 \| 32 \| 33 \| 35 \| 36 \| 37 \|   • **The findings of this study suggests that the lower 95% CI of normal MAP in neonates of 23**–**35 w is approximately 2 mmHg lower than their GA in the 1^st^ 12 h of life and increases by 1**–**2 mmHg in neonates of** ≤  **30 w and 3**–**4 mmHg in neonates of >  30 w.** |
| Lee 2012  Canada  Retrospective cohort study | < 27 | NI | < 7 d | This study evaluated the association of hypotension with three groups of neonates:  G1: Neonates with no morbidities or mortality.  G2: Neonates with one or two morbidities but no mortality.  G3: Neonates who had either all three morbidities (IVH>  grade 2, severe ROP, BPD defined as O_2_ or respiratory support requirement at 36 weeks’ PMA) or who died before discharge. | • Definition of hypotension: MAP < GA  • GA cut-off: <  29 w  • NIBP or IBP was used  • **MAP values were similar in all three groups indicating that isolated hypotension defined as MAP < GA was not associated with poor short term outcomes or mortality** |
| LeFlore 2000  Ireland  Prospective cohort study | – | VLBW | <72 h | The primary objective of the study was to examine the effect ANS on systolic BP, diastolic BP and MAP (ANS exposed, n = 70; ANS unexposed = 46). | • NIBP and IBP were used.  • **Volume replacement and/or inotropes were administered at the clinician’s discretion. The existing unit protocol for intervention was when there was evidence of poor perfusion (capillary refill> 3s) and/or MAP < 25–30 mmHg.**  • **Of the 105 neonates ≤  1500 g who did not receive treatment for ‘hypotension,’ the incidence of IVH>  grade 2 did not differ between those in whom at least one MAP was less than 30 mmHg and those in whom all MAPs were >  30 mmHg, (P =  0.67).**  •The incidence of any grade IVH did not differ between neonates who received therapy and who did not.  • Only BP data obtained prior to the interventions were used in analyses.  • The authors concluded that ANS exposure was not associated with improved systolic BP, diastolic BP and MAP. |
| Liu 2022  China  Prospective cohort study | I: 30.2 ± 1.6  C: 29.2 ± 1.7 | I: 1412 ± 321  C: 1294 ± 288 | < 72 h | The study compared two MAP thresholds for treatment of hypotension with or without clinical/ biochemical signs of hypoperfusion. The primary aim of the study was to determine the most appropriate MAP cut-off for treatment of hypotension:: MAP < GA (G1, n = 104) vs. MAP <  30 mm Hg (G2, n = 114). | • Definitions of hypotension that were compared: MAP < GA (G1) vs. MAP <  30 mm Hg (G2)  • The authors reported that neonates in both the groups had higher incidence of signs of poor perfusion, hs PDA and pulmonary hypotension when compared to normotensive control group  • **Based on multivariate logistic regression the authors concluded that hypotension based on either of the definitions was not an independent risk factor for poor prognosis**  • Whilst G1 had 58.7% with signs of poor perfusion, G2 had 55.3% with signs of poor perfusion |
| Limperopoulos 2007  U.S.A.  Prospective cohort study | 26 (23–30) | 880 (460–1490) | < 72 h | The objective of this study was to apply MAP values prospectively collected from their cohort of neonates and apply to 3 commonly used definitions of hypotension in current clinical use, and derive a hypotensive index for each definition (n = 84). | • 3 Definitions compared were MAP <  30 mm Hg, MAP < GA and MAP < 10^th^ percentile of MAP for BW and postnatal age based on published normative data  • GA cut-off: <  30 w  • Post hoc a 4^th^ definition of MAP <  33 mm Hg (10^th^ centile from this studies’ cohort) was also analysed  • In this study hypotension was treated based on clinical as well biochemical criteria as well  • The authors concluded that all the three definition utilized were not associated with brain injury and that treatment based on any of these definitions may not prevent brain injury |
| Liping 2023  China  Retrospective case control study | <28 | – | <72 h | The primary objective of the study was to assess the risk factors and prognosis associated with hypotension in the first 72 h of life in ELGANs. Two groups were compared: hypotension group (n = 41) and normotension group (n = 82). | • IBP and NIBP were used.  • There were significant differences in the baseline characteristics between the two groups with the hypotensive group being sicker.  • Definition of hypotension was MAP < GA  • Only unadjusted analyses of the various outcomes were given.  • Hypotensive group had significantly higher risk of morbidities including PDA requiring treatment, IVH>  grade 2, pulmonary hemorrhage and mortality within 7 d of life (unadjusted for baseline sickness). |
| Logan 2011  U.S.A  Prospective cohort  Multi-centre study | < 28 | – | < 24 h | The study compared three approaches along with MAP values for treatment of isolated hypotension and their association with neonatal outcomes:  A. Lowest MAP in the lowest quartile for gestational age (23–24, 25–26 and 27 weeks, n = 219)  B. Treatment for hypotension with a vasopressor (dopamine, dobutamine  or epinephrine) (n = 250).  C. Blood pressure lability, defined as the upper quartile of the difference between the lowest and highest MAP (n = 250). | • Definition of hypotension: As specified in the previous column. The neonates treated predominantly had isolated hypotension with no clinical signs or symptoms of hypotension  • NIBP or IBP was used  • **The authors reported that after adjusting for confounders, no association was found between any of the three indicators of hypotension and white matter damage or CP, The aOR were depicted in a figure and the exact values were not discernible**  • **The authors concluded that early isolated hypotension may not be associated with brain injury in ELGANs** |
| Martens 2003  Netherlands  Prospective cohort study | 29.2 (23.4–31.6) | 1250 (383) | NI | This study aimed to evaluate the association of isolated hypotension with brain injury at term equivalent gestation in preterm neonates of less than 32 weeks’ GA (n = 211). | • Definition of hypotension: MAP <  30 mm Hg on at least 2 occasions  • NIBP or IBP were utilized  • GA cut-off: <  32 w  • **When adjusted for PVL and other confounders, isolated hypotension was not associated with abnormal neurological examination (Prechtl’s method) [aOR of 1.87 (95% CI 0.94– 3.71)] at term gestation equivalent** |
| Miall-Allen 1987  London  Prospective cohort study | 27.7 (26–30) | 1060 (700–1600) | < 48 h | Serial monitoring of IBP was done and the association of MAP with brain injury and mortality was studied. Pethidine and pancuronium were given when required, and plasma or 5% human albumin was given slowly(10–15ml/kg) if perfusion was considered inadequate. Of the 33 neonates enrolled, 7 were treated with inotropes. | • Treatment of hypotension was based on clinician’s discretion  • IBP was used  • GA cut-off: <  31 w  • **The authors reported that MAP of <  30mm Hg for over an hour was significantly associated with severe haemorrhage, ischaemic cerebral lesions, or death within 48 h. No severe brain lesions developed with a MAP ≥  30 mm Hg**  • **The treatment for cardiovascular insufficiency was based on clinician’s discretion and it was not reported how many of the neonates with MAP ≤  30 mm Hg had signs of poor perfusion. This makes it difficult to interpret whether major brain injury was associated with isolated hypotension or clinical hypotension** |
| Meek 1999  U.K.  Prospective observational study | <32 | – | < 24 h | The objective of this study was to evaluate the CBF on the first day of life and its association with severity of IVH. NIRS was used to measure the CBF (n = 24). | • No information provided regarding the definition of hypotension or its management  • **The authors reported that those neonates with lowest CBF [5.8 (3.2–7.5) ml/100 g min)] had severe IVH ( > grade 2) when compared to those with a higher CBF [12.1 (6.1–24.7) ml/100 g/ min].**  • **Those neonates with lowest CBF had significantly higher MAP in the first 24 h of life [35.0 (35–43) mm Hg] when compared to those with higher CBF [29.5 (25–40) mm Hg]. A reasonable inference that could be drawn from this finding is that increased MAP in the first 24 h of life might be associated with severe grade IVH** |
| Pellicer 2009  U.S.A  Retrospective case control study | <32 | <1501 | <24 h | Cases included neonates who were treated with either dopamine or epinephrine for hypotension (n = 60). The control group included normotensive neonates (n = 70). | • Hypotension was defined as MAP lower than GA that persisted at least for 60 minutes at any time in the first 24 h of life.  • Both IBP and NIBP monitoring was used.  • Optimal MAP was defined as a 15% increase over the corresponding lower limit of MAP established for each neonate according to GA.  • Dose of dopamine was increased in a stepwise manner from 2.5 to 10 mcg/kg/min whereas the dose of epinephrine used was 0.125 to 0.50 mcg/kg/min.  • Multivariate analysis did not detect any association between final cranial ultrasounds and the use of vasopressors/inotropes.  • No differences between groups were found in the rates of abnormal neurologic status, developmental delay, or combined adverse outcome (death or cerebral palsy or severe neurodevelopmental delay) (unadjusted analysis)  • Though the control group were less sicker compared to the treatment group, long-term outcomes were similar.  • The authors concluded that inotropes should be used with caution in isolated hypotension in the first 24 h of life. |
| St Peter 2017  U.S.A  Retrospective cohort study | <29 | – | < 72 h | The aim of the study was to compare two definitions of hypotension and their association with neonatal mortality: MAP < GA and MAP <  30 mm Hg. Treatment with inotropes was initiated when MAP < GA with clinical signs of hypoperfusion (n = 376) | • Two definitions of hypotension were compared as specified prior  • NIBP or IBP was used  • GA cut-off: <  29 w  • **Multi-variate regression analysis indicated that neither of the definitions predicted the combined outcome of mortality or severe IVH - However, only GA and vasopressor support (initiated based on MAP cut off with clinical signs of poor perfusion) were predictors of the composite outcome.** |
| Trounce 1988  U.K.  Retrospective cohort study |  | <1500 | NI | Multiple risk factors for severe brain injury based on multi-variate logistic regression (n = 200) | • Details related to hypotension such as definition, treatment criteria and postnatal age till BP were monitored were not specified  • NIBP or IBP was used  • The authors concluded that there was no relation between hypotension or the development of PVL based their multivariate regression analysis correcting for baseline sickness |
| Victor 2006  U.K.  Prospective observational study | 27 (23–30) | 935 (470–1495) | < 48 h | This study evaluated the effect of different levels of MAP on PBF, CFOE NIRS and cerebral electrical activity using aEEG (n = 35) | • The unit practice was to maintain MAP >  10^th^ centile, the attending physician was not a part of the research team  • GA cut-off: <  30 w  • The EEG became abnormal at MAP <  23 mm Hg  • Abnormally high CFOE was measured at 20 mm Hg  • PBF decreased at MAP range of 23–33 mm Hg  • The authors concluded that cerebral perfusion is probably maintained at MAP levels above 23 mm Hg |
| Watkins 1998  U.K.  Retrospective cohort study | 28 (24 - 34) | 1050 (558–1500) | < 96 h | This retrospective cohort study evaluated the association of hypotension in the first 96 h of life and any grade IVH (n = 131) | • Hypotension was defined as MAP <  10^th^ centile for GA, BW and postnatal age for >  2 consecutive readings  • IBP was used  • **Unadjusted analysis indicated that hypotension was associated with any grade IVH and that hypotension episodes were observed the day before as well the day after the occurrence of IVH. Hence, the authors also concluded that IVH may have given rise to the hypotension or the vice versa.** |
| Weindling 1985  U.K.  Prospective cohort study | < 34 | ≤1500 | ≤ 5 d | The objective of the study was to determine the risk factors for PVL | • Definition of hypotension was MAP <  30 mm Hg on a single occasion or if signs of poor perfusion was observed irrespective of MAP  •IBP was used  • **The authors found no association between hypotension and PVL (univariate regression)** |
|  | | | | | |

GA: Gestational age, BW: Birth weight, w: Weeks, g: Grams, I: Intervention group, C: Control group, G1: Group 1, G2: Group 2, G3: Group 3, SD: Standard deviation, IQR: Interquartile range, RCT: Randomized controlled trial, h: hours, d: day, D: Definition, BP: Blood Pressure, RDS: Respiratory distress syndrome, IVH: Intraventricular hemorrhage, BPD: Bronchopulmonary dysplasia, NEC: Necrotising enterocolitis, ROP: Retinopathy of prematurity, PDA: Patent ductus arteriosus, PVL: Periventricular leukomalacia, PVH: Peri-ventricular hemorrhage, MAP: Mean arterial pressure, CFT: Capillary refilling time, IBP: Invasive BP, NIBP: Non-invasive BP, ELGANs: Extremely low gestational age neonates, ELBW: Extremely low birth weight, VLBW: Very low birth weight, EEG: Electroencephalogram, aOR: Adjusted odds ratio, 95% CI: 95% confidence interval, NDI: Neurodevelopmental impairment, CP: Cerebral palsy, CA: Corrected age, MDI: Mental development index, PDI: Psychomotor development index, CBF: Cerebral blood flow, NIRS: Near infra-red spectroscopy, CFOE: Cerebral fractional oxygenation extraction, PBF: Peripheral blood flow, ANS: Antenatal corticosteroids, NA: Not applicable, NI: No information

*Intervention group: Treatment with volume expansion and/ or inotropes for Isolated hypotension. Isolated hypotension was defined as low MAP as ascertained by the investigator which could be based on definitions such as MAP less than gestational age, MAP value below the 5^th^ centile for the particular gestational age, MAP less than 30 mm Hg without any clinical or biochemical evidence of hypoperfusion.

*Control group:

a. Treatment with volume expansion and/ or inotropes in preterm neonates with hypotension only when clinical and/ or biochemical features of poor perfusion were present. The clinical and/ or biochemical signs of hypoperfusion were defined as follows: Tachycardia (>160–170 beats/min), prolonged capillary refilling time ( > 3–4 seconds), low peripheral pulses, decreased urine output ( < 1 ml/kg/ hour for 4–6 hours, physiological oliguria or anuria should also be considered), increasing lactate levels ( > 3–4 mmol/L) and base deficit ( > 8 meq/L).

b. No treatment of isolated hypotension.
