# Supplementary material for: Active versus restrictive approach to isolated hypotension in preterm neonates: A Systematic Review, Meta-analysis and GRADE based Clinical Practice Guideline
Source: PLoS One. 2025 Mar 18;20(3):e0309520. doi: 10.1371/journal.pone.0309520 (PMC11918419; doi:10.1371/journal.pone.0309520)
Supplement: Attachment — Submitted filename: response_to_reviewers.docx [file pone.0309520.s005.docx]

**Response to Editorial Board and Reviewer comments:**

1. When submitting your revision, we need you to address these additional requirements.

Please ensure that your manuscript meets PLOS ONE's style requirements, including those for file naming. The PLOS ONE style templates can be found at 
<https://journals.plos.org/plosone/s/file?id=wjVg/PLOSOne_formatting_sample_main_body.pdf> and 
<https://journals.plos.org/plosone/s/file?id=ba62/PLOSOne_formatting_sample_title_authors_affiliations.pdf>.

**Response:**

*We have formatted the manuscript as per PLOS ONE’s requirements.*

2. Please amend either the title on the online submission form (via Edit Submission) or the title in the manuscript so that they are identical.

**Response:**

*We have addressed this aspect.*

3. Please amend the manuscript submission data (via Edit Submission) to include author Abdul Kareem Pullattayil S.

**Response:**

*We have amended the manuscript submission data.*

4. One of the noted authors is a group [National Neonatal Forum (NNF), Clinical Practice Guidelines (CPG) Group, India]. In addition to naming the author group, please list the individual authors and affiliations within this group in the acknowledgments section of your manuscript. Please also indicate clearly a lead author for this group along with a contact email address.

**Response:**

*We made the suggested changes. (Page 13 -14, Line 285 – 308)*

5. Please include your tables as part of your main manuscript and remove the individual files. Please note that supplementary tables (should remain/ be uploaded) as separate ""supporting information"" files".

**Response:**

*We have embedded the three tables within the manuscript. The supplementary file has been uploaded as ‘supporting information file’.*

6. Please include captions for your Supporting Information files at the end of your manuscript, and update any in-text citations to match accordingly. Please see our Supporting Information guidelines for more information: <http://journals.plos.org/plosone/s/supporting-information>.

**Response:**

*We have made the suggested changes (Page 20).*

Reviewers' comments:

Reviewer's Responses to Questions

**Comments to the Author:**

1. Is the manuscript technically sound, and do the data support the conclusions?

The manuscript must describe a technically sound piece of scientific research with data that supports the conclusions. Experiments must have been conducted rigorously, with appropriate controls, replication, and sample sizes. The conclusions must be drawn appropriately based on the data presented.

Reviewer #1: Yes

Reviewer #2: No

2. Has the statistical analysis been performed appropriately and rigorously?

Reviewer #1: Yes

Reviewer #2: No

3. Have the authors made all data underlying the findings in their manuscript fully available?

The [PLOS Data policy](http://www.plosone.org/static/policies.action#sharing) requires authors to make all data underlying the findings described in their manuscript fully available without restriction, with rare exception (please refer to the Data Availability Statement in the manuscript PDF file). The data should be provided as part of the manuscript or its supporting information, or deposited to a public repository. For example, in addition to summary statistics, the data points behind means, medians and variance measures should be available. If there are restrictions on publicly sharing data—e.g. participant privacy or use of data from a third party—those must be specified.

Reviewer #1: Yes

Reviewer #2: Yes

4. Is the manuscript presented in an intelligible fashion and written in standard English?

PLOS ONE does not copyedit accepted manuscripts, so the language in submitted articles must be clear, correct, and unambiguous. Any typographical or grammatical errors should be corrected at revision, so please note any specific errors here.

Reviewer #1: Yes

Reviewer #2: Yes

**Response to the Reviewers:**

*We thank the Reviewers for their critical appraisal of our manuscript. We have modified the manuscript in accordance with their comments and suggestions.*

5. Review Comments to the Author

Please use the space provided to explain your answers to the questions above. You may also include additional comments for the author, including concerns about dual publication, research ethics, or publication ethics. (Please upload your review as an attachment if it exceeds 20,000 characters)

Reviewer #1: This is good review on the topic Active versus Restrictive management of Isolated Hypotension in preterm infants. Although the evidence evaluated did not result in strong recommendations, the authors suggest the need for well conducted RCT on this important topic.

**Response to the Reviewer #1:**

*We thank the Reviewer for the comments.*

I feel the paragraphs on the narrative review may be deleted altogether to improve the readability of the study. However some sections of the narrative review may be used by the authors in the discussion to understand how some the weak or expert recommendations were arrived at.

**Response to the Reviewer:**

*We thank the Reviewer for these suggestions.*

*Accordingly, we have moved the ‘Narrative review’ section to ‘S1_File’ and labelled the section as ‘Appendix’.*

*We would also like to bring to the esteemed Reviewers’ attention that we have provided the complete evidence to decision (EtD) framework as per the GRADE working group guidelines in ‘S4 Table’ in ‘S1_File’. Since these recommendations were based on both quantitative meta-analyses and descriptive review of studies that could not be included in the data synthesis, we decided to provide whole EtD framework along with the justifications for providing weak recommendations. We have also indicated this in the manuscript in the discussion section as suggested by the Reviewer. The readership could go in detail the process that was involved in the framing of these recommendations in the EtD framework as indicated in the manuscript. (Page 10, Lines 209-214)*

**Response to the Reviewer #2:**

Reviewer #2: Active Versus Restrictive Approach to Isolated Hypotension In Preterm Neonates: A Systematic Review And Meta-analysis

The authors tried to address an important yet les studied and controversial topic. They have systematically approached the topic and must be congratulated for their efforts.

**Response to the Reviewer:**

*We thank the Reviewer for the encouraging comments.*

Here are certain observations which might be of help in revising the manuscript:

1. The authors must define what does active and restrictive approach mean. Throughout the manuscript there is no mention of these two strategies.

**Response to the Reviewer:**

*We thank the Reviewer for this suggestion. We had defined both the approaches in the ‘inclusion criteria’ sub-section (Page 4, Lines 83-100). As you are well aware hypotension was defined varyingly by the authors of the included studies. The following definitions have been commonly used for hypotension in VPT infants: Mean arterial blood pressure (MAP) less than the gestational age, MAP based on centiles, MAP < 30 mm Hg etc. Hence, for such a less studied and debatable topic, we decided to define hypotension based on the primary authors’ / investigators’ definition. We have amended the inclusion criteria by defining isolated hypotension (response to the Reviewer query 3) and the clinical and / or biochemical features of hypoperfusion (the clinical / biochemical criteria for defining hypoperfusion was already mentioned in S4 Table under the sub-section ‘implementation considerations’). We amended the definitions to bring in more clarity in the modified manuscript:*

*Intervention(I) (active treatment group): Treatment with inotropes for IH. Volume expansion with crystalloids or colloids could have been used prior to inotrope or vasopressor initiation. (Page 4, Lines 89-91)*

*Comparator(C) (restrictive treatment group):*

*a. Treatment with volume expansion and / or inotropes in preterm neonates with hypotension only when clinical and / or biochemical features of poor perfusion were present. The clinical and / or biochemical signs of hypoperfusion were defined as presence of either of these: Unexplained tachycardia (>160-170 beats/min), prolonged capillary refilling time (> 3-4 seconds), low peripheral pulses, decreased urine output (< 1 ml/kg/ hour for 4 -6 hours, physiological oliguria or anuria should also be considered), increasing lactate levels (> 3-4 mmol/L) and base deficit (> 8 meq/L).*

*b. No treatment of IH. (Page 4, Lines 92 – 100)*

2. The authors must describe the active and control group strategies in table 1 and table 2. Without clear description one will not know whether they are comparable.

**Response to the Reviewer:**

*We thank the Reviewer for the valuable suggestions. We have included the two approaches (as modified according to the previous suggestion by the Reviewer) in the footnotes in Table 1 and Table 2. (Pages 26 and 38)*

3. Inclusion criteria: Population is not well defined. The population definition should contain preterm infants with isolated hypotension. This isolated hypotension must be elaborated well like by what definition the diagnosis should be made. As there will be huge variation in the way the condition is defined, and it will have direct impact on the results.

**Response to the Reviewer:**

*We thank the Reviewer for the suggestions. We have added ‘isolated hypotension’ in the population. The definition of isolated hypotension has also been mentioned. We had a priori defined in our protocol that the MAP cut-off for IH would be ‘as defined by the authors’*. *The following changes have been made:*

*“Population(P): Preterm neonates of less than 37 weeks’ gestational age within the first week of life with IH. IH was defined as low mean arterial blood pressure (MAP) as ascertained by the investigator which could be based on definitions such as MAP less than gestational age, MAP value below the 5th centile for the particular gestational age, MAP less than 30 mm Hg without any clinical or biochemical evidence of hypoperfusion.”(Page 4, Lines 84 - 88)*

4. Comparator also needs clarity. Which gain takes us back to defining the isolated hypotension.

**Response to the Reviewer:**

*We thank the Reviewer for the comment. This section has been revised as per Reviewer comment 1. (Page 4, Lines 92 – 100)*

**5.** Literature search: The search was limited to only 2 databases. As the authors said that this topic is very controversial with limited literature, it becomes more pertinent to include at least 3 databases. Also, it will be better if the search is updated till end of the year.

**Response to the Reviewer:**

*We thank the Reviewer for the suggestions. We have updated the literature search of the data bases Medline and Embase until 1^st^ April 2024. We have also included another database Web of Science and done a systematic literature search of WOS until 1^st^ April 2024 as well. Accordingly, changes have been made to the PRISMA flow (Figure 1), abstract section (Page 2), literature search strategy section (page 5, line 114), results section (Page 6, Lines 140-146), S1_File: S1b Table and the narrative review of the studies (S1 File: Narrative review). We could include 7 recently published additional studies (References: 46 – 52).*

6. The authors stated that the definition of IH varied. The variation is so much that it might not be wise to pool them. Particularly if some of the studies did not use BP as a measure to define IH.

**Response to the Reviewer:**

*We thank the Reviewer for the suggestion. Most studies have used BP as a measure of isolated hypotension. The two RCTs had taken almost similar (if not exactly the same definitions). The results of our meta-analyses also did not reveal any statistical heterogeneity. Hence, we feel that meta-analyses of these two small sized RCTs may possibly be justified.*

*Coming to observational studies, Faust et al., Aladangady et al., Durrmeyer et al., Kuint et al., Gogcu et al. have all used quite similar definitions to define hypotension which was MAP < GA. Only Dammann et al. had used a different definition (quartile based). We included Dammann et al. study as we presumed the definition used would be quite similar to that of Faust et al. as the BP centiles in Dammann et al. were equivalent to MAP < GA. Batton et al. had used a divergent definition for IH and we did not include the study in the meta-analysis. We were quite conscious of this important aspect pointed out by the Reviewer before proceeding ahead with the meta-analyses. Also, we had addressed this issue by downrating the evidence for indirectness related to definition of IH (population) wherever appropriate (Table 3, explanation ‘b’)(page 42)*

7. Outcomes: The authors should provide the risk estimates in text also particularly for important outcomes rather than just referring to Tables and figures.

8. At all places , the number of studies with number of participants must be mentioned along with effect estimates. This provides a wholesome view to the reader. Many of the outcomes are from a handful of participants.

**Response to the Reviewer query 7 and 8:**

*We thank the Reviewer for these suggestions. We have given the effect estimates with 95% CI for all the outcomes along with the number of studies and the sample size. (Pages 8 – 10)*

9. Dempsey 2021 compared fluid+ inotrope vs fluid only. How come the first is active approach and second is restrictive

10. Pereira 2019 USA 3-armed pilot RCT: In this study all received fluid bolus and inotropes though at different cut-offs. So technically it should not be combined with Dempsey 2021.

**Response to the Reviewer query 9 and 10:**

*We thank the Reviewer for the query. We would like to state that boluses have been given in many studies including the ones pointed out by the author. As stated in response to Reviewer comment 6, our initial scoping review did bring out these aspects and we decided to do a pragmatic meta-analyses of studies that had quite similar but not exactly the same PICO. As stated before we did not find any significant heterogeneity when pooling the data from these studies. Likewise for the patient population (definition of IH), we had downgraded the evidence for indirectness related to intervention / comparator. As you are well aware, as a Clinical practice Guideline Group we had to make recommendations with the best available evidence that could be generalizable to different settings. Henceforth, we presumed that a pragmatic rather than an explanatory meta-analyses would possibly be the appropriate way forward. The evidence certainty from the meta-analyses of these two RCTs is ‘very low’ now. We have updated them in the manuscript as well. We have included these in the limitations section:*

*“There were several limitations to this meta-analysis. The definition of isolated hypotension and clinical hypotension with signs of hypoperfusion varied between studies. Further, some of the neonates in the restrictive approach group received volume expansion which could have altered the effect estimates of the meta-analyses. We tried to address these by downrating the evidence by one level for the domain of indirectness related to patient population and the intervention / comparator.****”*** *(Page 12, Lines 269 - 275)*

*We took the decision to do a meta-analysis with a pragmatic approach based on the suggestion by the GRADE group:*

“First, if results prove similar from study to study, the variability in study PICOs enhances the generalizability of those results. If patient populations differ widely and results are nevertheless similar across studies, it tells us that patients with different characteristics respond similarly to the intervention, allowing application of the results to a wide range of patients. If studies use different doses but results are nevertheless similar across studies, this tells us intervention effects are independent of dose. Clinicians can then choose freely from among the doses used in the studies (e.g., use lower doses that are less likely to cause adverse effects). Similar interpretations would apply to differences in the comparator and outcomes.”

Reference: <https://pubmed.ncbi.nlm.nih.gov/36898507/>

*In our study, there were differences in “PIC” between the studies, the meta-analyses of most of the outcomes revealed almost similar results. Wherever, significant heterogeneity was detected, we did sub-group analyses and found the postnatal age of enrolment to be a significant factor contributing to the heterogeneity. When we extrapolate the GRADE group guidance to our systematic review, we could reasonably infer the following:*

A. *Irrespective of the definition of IH used, the restrictive approach might possibly result in better outcomes.*
B. *These results are generalizable to NICUs where volume expansion alone is used (and not inotropes or vasopressors) for treating IH (restrictive approach).*

11. The active group of Batton 2016 has significant overlap with Dempsey 2021.

**Response to the Reviewer:**

*We thank the Reviewer for the observation. The BP values utilized by the two studies were different. Henceforth, we couldn’t include the Batton 2016 study in the meta-analyses for any of the outcomes.*

12. How did the authors assess inconsistency? I hope it was limited to statistical heterogeneity.

**Response to the Reviewer:**

*We thank the Reviewer for the query. We assessed inconsistency based on multiple parameters, not restricted to I^2^ alone. We followed the GRADE working group guidelines on inconsistency assessment.* *(https://pubmed.ncbi.nlm.nih.gov/21803546/).*

*As you are aware, GRADE emphasizes on statistical heterogeneity. We followed that for inconsistency assessment. The following were considered while adjudging inconsistency: the similarity of point estimates, overlap of confidence intervals, the statistical test of heterogeneity, and the I^2^.*

13. The GRADE profile does not seem to be consistent with recent GRADE recommendations. At many places , especially for imprecision the down gradation should be by 2-3 points. Particularly for RCTs where the cumulative sample size is < 400 and CI are extremely wide.

**Response to the Reviewer:**

*We thank the Reviewer for pointing this important aspect. We had utilized the older version of GRADE guidelines for rating imprecision. We have amended it in the present manuscript. (Table 3, domain ‘imprecision’). We have evaluated imprecision in the modified manuscript based on the confidence interval approach (as suggested by the Reviewer) using a minimally contextualized approach*

*Reference: GRADE guidance 34 paper “GRADE Guidance 34: update on rating imprecision using a minimally contextualized approach”*

*Further, the sample size suggested by the Reviewer is for continuous outcomes and all of the outcomes in this systematic review were dichotomous outcomes. For continuous outcomes, the GRADE guidance 34 paper (page 223) suggests the following:*

“Using the OIS in the context of continuous outcomes presents complexities that do now allow creating guidance based on the boundaries of the CI. If authors are not, however, confident of the MID or the standard deviation (SD) needed for the calculation of OIS, they could use an effect size of 0.2 SDs that represents a small effect [13]. This results in a total sample size of approximately 800 (400 per group) [1]. GRADE suggests, based on OIS approach, rating down two levels for imprecision if the total sample size of meta-analysis is smaller than arbitrary threshold of 30 - 50% of OIS (Box 5, circumstance 2). If the authors choose to be more conservative, they could choose 50% of OIS as a threshold (i.e., 400 overall); if they choose to be less conservative, they could use 30% of OIS as the threshold (i.e., 240 overall).”

14. Was Pereira 2019 really at low risk of bias in all domains. It was retrospectively registered with no protocol available at website. Intervention is open label, and many outcomes are subject to observer bias.

**Response to the Reviewer:**

*We thank the Reviewer for the query. As the Reviewer is aware, RoB 2.0 is a subjective tool and may be interpreted differently. As far as the Pereira et al. study is concerned, we adjudged it as to have an overall low risk of bias. The two domains the Reviewer was concerned about were blinding and selective reporting. Most of the critical outcomes based on which this CPG was formulated were objective ones, eg. mortality. So, according to the signalling question, point 4.4 in the figure below we presumed that the assessment of these outcomes may not be influenced by the knowledge of the intervention. Hence, we rated this domain as having a low risk of bias.*

*The second domain that the Reviewer had concerns was selective reporting and the retrospective registration of the protocol. Our group as per the Cochrane Neonatal has always been less parsimonious in evaluating this domain. If the authors had reported most of the critical and important outcomes, we rated the domain as low risk. However, as evident from the figure below, if we were to follow the signalling questions stringently as rightly suggested by the Reviewer, this domain will be rated as ‘some concerns’. Since we have used an uniform approach for rating this domain for all the studies (reason being many of the included studies, especially the observational studies were published years before RoB 2.0 / ROBINS-i tools were introduced and a priori registration of the protocol was not mandatory during that time period). Finally, even if we make the overall risk of bias for this RCT as ‘some concerns’, the evidence certainty will still remain very low for all the outcomes. We are happy to modify if the Reviewer feels otherwise.*

15. Many observational studies do not actually meet the criteria for inclusion. They are hugely different from each other with significant overlap between two groups among themselves and with other studies.

**Response to the Reviewer:**

*We thank the Reviewer for the comments. Our objective was to frame a clinical practice guideline based on the GRADE recommendations. In the EtD framework, we had to include all the possible studies supporting the primary studies which were included. Hence, we included all the observational studies relevant to the CPG. As indicated by the esteemed Reviewer, these were supporting evidence as indicated in the S4 Table.*

Based on above observations the authors should rethink whether the meta-analysis should be done.

**Response to the Reviewer:**

*We thank the Reviewer for the suggestion. We had responded to this Reviewer comment in the response to Reveiwer queries 9 and 10 as to why decided to meta-analyses of some studies. We also based our judgement as guided by the GRADE recommendations:*

“Clinicians themselves will rarely explore the evidence as thoroughly as a guideline panel, nor will they devote as much thought to the trade-offs, or the possible underlying values and preferences in the population. GRADE encourages panels to deal with their discomfort and to make recommendations even when confidence in effect estimate is low and/or desirable and undesirable consequences are closely balanced. Such recommendations will inevitably be weak, and may be accompanied by qualifications.”

*Reference:* [*https://gdt.gradepro.org/app/handbook/handbook.html#h.zh3vgx3nht7m*](https://gdt.gradepro.org/app/handbook/handbook.html#h.zh3vgx3nht7m)

*Hence, to arrive at recommendations for this PICO we needed to do take a pragmatic approach and synthesize data from studies that were not very divergent. From the results of the meta-analyses of these studies for the various outcomes we could go through the EtD framework and finally come out with recommendations which were weak with the evidence certainty being very low. We hope this manuscript will pave way to future studies on this less explored PICO.*

Yours Sincerely

On Behalf of Author Team

Viraraghavan Vadakkencherry Ramaswamy
