# Supplementary material for: Active versus restrictive approach to isolated hypotension in preterm neonates: A Systematic Review, Meta-analysis and GRADE based Clinical Practice Guideline
Source: PLoS One. 2025 Mar 18;20(3):e0309520. doi: 10.1371/journal.pone.0309520 (PMC11918419; doi:10.1371/journal.pone.0309520)
Supplement: Attachment — Submitted filename: response_to_reviewers_2.docx [file pone.0309520.s006.docx]

Dear Dr. More,

Thank you for submitting your manuscript to PLOS ONE. After careful consideration, we feel that it has merit but does not fully meet PLOS ONE’s publication criteria as it currently stands. Therefore, we invite you to submit a revised version of the manuscript that addresses the points raised during the review process.

Please submit your revised manuscript by Jul 14 2024 11:59PM. If you will need more time than this to complete your revisions, please reply to this message or contact the journal office at [plosone@plos.org](mailto:plosone@plos.org). When you're ready to submit your revision, log on to <https://www.editorialmanager.com/pone/> and select the 'Submissions Needing Revision' folder to locate your manuscript file.

Please include the following items when submitting your revised manuscript:

- A rebuttal letter that responds to each point raised by the academic editor and reviewer(s). You should upload this letter as a separate file labeled 'Response to Reviewers'.
- A marked-up copy of your manuscript that highlights changes made to the original version. You should upload this as a separate file labeled 'Revised Manuscript with Track Changes'.
- An unmarked version of your revised paper without tracked changes. You should upload this as a separate file labeled 'Manuscript'.

We look forward to receiving your revised manuscript.

Kind regards,

Sanjoy Kumer Dey, M.D
Academic Editor
PLOS ONE

***Response to the Editor:***

*Dear Sanjoy K Dey, M.D*

*Academic Editor*

*PLOS ONE*

*We would like to thank you for giving us the opportunity to address the Reviewer’s comments and suggestions. We also would like to thank the PLOS ONE team for ensuring a timely re-review of our manuscript.*

*Sincerely Yours*

*Viraraghavan V Ramaswamy*

*On behalf of the Author Team and National Neonatology Forum, India CPG 2023 group*

Reviewers' comments:

Reviewer's Responses to Questions

**Comments to the Author**

1. If the authors have adequately addressed your comments raised in a previous round of review and you feel that this manuscript is now acceptable for publication, you may indicate that here to bypass the “Comments to the Author” section, enter your conflict of interest statement in the “Confidential to Editor” section, and submit your "Accept" recommendation.

Reviewer #1: All comments have been addressed

Reviewer #2: (No Response)

***Response to Reviewer #1:*** *We thank the Reviewer for the comment.*

2. Is the manuscript technically sound, and do the data support the conclusions?

The manuscript must describe a technically sound piece of scientific research with data that supports the conclusions. Experiments must have been conducted rigorously, with appropriate controls, replication, and sample sizes. The conclusions must be drawn appropriately based on the data presented.

Reviewer #1: Yes

Reviewer #2: Partly

***Response to Reviewer #1 and Reviewer #2:*** *We thank the Reviewers for accepting most of the suggested changes that we had made in the modified manuscript and for the point by point response given to the Reviewer comments.*

3. Has the statistical analysis been performed appropriately and rigorously?

Reviewer #1: Yes

Reviewer #2: No

***Response to Reviewer #1 and Reviewer #2:*** *We thank the Reviewers for their comments We would like to elaborate with regards to this question to Reviewer #2 comment 6 in the forthcoming section.*

4. Have the authors made all data underlying the findings in their manuscript fully available?

The [PLOS Data policy](http://www.plosone.org/static/policies.action#sharing) requires authors to make all data underlying the findings described in their manuscript fully available without restriction, with rare exception (please refer to the Data Availability Statement in the manuscript PDF file). The data should be provided as part of the manuscript or its supporting information, or deposited to a public repository. For example, in addition to summary statistics, the data points behind means, medians and variance measures should be available. If there are restrictions on publicly sharing data—e.g. participant privacy or use of data from a third party—those must be specified.

Reviewer #1: Yes

Reviewer #2: Yes

***Response to Reviewer #1 and Reviewer #2:*** *We thank the Reviewers for the comments.*

5. Is the manuscript presented in an intelligible fashion and written in standard English?

PLOS ONE does not copyedit accepted manuscripts, so the language in submitted articles must be clear, correct, and unambiguous. Any typographical or grammatical errors should be corrected at revision, so please note any specific errors here.

Reviewer #1: Yes

Reviewer #2: Yes

***Response to Reviewer #1 and Reviewer #2:*** *We thank the Reviewers for the comments.*

6. Review Comments to the Author

Please use the space provided to explain your answers to the questions above. You may also include additional comments for the author, including concerns about dual publication, research ethics, or publication ethics. (Please upload your review as an attachment if it exceeds 20,000 characters)

Reviewer #1: the authors have made all the changes as suggested. The narartive review included in the appendix. Readability improed. No further changes are recommended

***Response to Reviewer #1:*** *We thank Reviewer #1 for the suggestions which had greatly improved the quality of the manuscript.*

Reviewer #2: Thank you considering my comments and revising the manuscript. However i still believe that the studies are not similar to combine them statistically.

***Response to Reviewer #2:*** *We thank Reviewer #2 for all the suggestions and critical appraisal of our initial manuscript. We had extensively revised our manuscript based on the critical inputs given by the Reviewer which made our literature search more comprehensive, the GRADE assessment rigorous, the definition of PICO clearer and finally, we could bring it out with more clarity to the readership that this was a pragmatic systematic review and not an explanatory one. We have detailed these aspects including why we synthesized the data with references from the GRADE working group and what are the implications of that in the revised manuscript.*

*Page 10-11, Lines 219- 238: “It should be noted that there was some clinical heterogeneity with regards to the patient population, the intervention and the comparator in the included studies in the meta-analyses for the various outcomes. The question of whether pooling of data in a meta-analysis arises in such scenarios. The GRADE working group provides clarity with respect to that. While GRADE visualizes the variability between studies as a source of potential opportunity. GRADE group points out that in some scenarios such variability might even turn out to be one of the strengths of a systematic review.(57) In our systematic review, the meta-analyses of RCTs did not show any between studies heterogeneity though there were some differences in the patient population, intervention and the comparator. This implies that the results of the meta-analysis of the RCTs could be generalizable to different clinical settings where isolated hypotension is defined based various definitions, the intervention and the comparator could deviate slightly still yielding similar clinical outcomes. Further, the meta-analyses of non-RCTs indicated significant heterogeneity for the critical outcome of mortality and the important outcome of severe grade IVH. This provided us an opportunity to explore the various potential reasons for the same. We found postnatal age to be an important effect modifier and hence we could bring out recommendations based on postnatal age of the neonate (≤24 h vs. > 24 h). Such pragmatic systematic reviews form the basis for formulation of clinical practice guidelines for difficult questions that often addressed through expert consensus. Further, these systematic reviews could be generalizable across different settings and also provide clinicians, parents and other stakeholders to choose between different treatment choices.”*
