# Supplementary material for: Active versus restrictive approach to isolated hypotension in preterm neonates: A Systematic Review, Meta-analysis and GRADE based Clinical Practice Guideline
Source: PLoS One. 2025 Mar 18;20(3):e0309520. doi: 10.1371/journal.pone.0309520 (PMC11918419; doi:10.1371/journal.pone.0309520)
Supplement: Attachment — Submitted filename: Respnse_R_3.docx [file pone.0309520.s007.docx]

Dear Sanjoy K Dey, M.D	5^th^ August 2024

Academic Editor

PLOS ONE

I thank you for giving us the opportunity to address the Reviewer’s comments and suggestions for the third revision of the manuscript. We have extensively addressed the queries raised by the previous Reviewers backed by published scientific literature on systematic reviews and clinical practice guidelines. Our group have would tried our best to address them in this third revision as well.

Sincerely Yours

Viraraghavan Vadakkencherry Ramaswamy

MD, DM (Neonatology), DNB (Neonatology) (LHMC, Delhi University, Delhi)

Neonatal trainee (OUH, Oslo, Norway)

Clinical Fellow (Oxford University Hospitals, U.K.)

Consultant Neonatologist and Head (Ankura Hospital for Women and Children, KPHB, Hyderabad, India) (2020- )

Associate Task Force Member, Neonatal Life Support, ILCOR (2021-)

Member, Cochrane Neonatal (2021-)

Guest faculty, KDIGO (2023)

Editorial Board Member, BMC Pregnancy and Childbirth (2023-)

Editorial Coordinator, National Neonatal Forum, India Clinical Practice Guidelines Group (2020-)

Editorial Board Member, Journal of Neonatology (2024-)

Member, Global Neonatal Resuscitation Alliance (2024-)

On behalf of the National Neonatology Forum, India Clinical Practice Guidelines Group 2023

**Response to the Reviewer**

Reviewer #2: Thank you for providing opportunity to review this manuscript. I have few observations

1. The definitions for intervention and control are hugely different across studies, hence might not be appropriate to combine them.

The following definitions have been commonly used for hypotension in VPT infants: Mean arterial blood pressure (MAP) less than the gestational age, MAP based on centiles, MAP < 30mm Hg etc. Hence, for such a less studied and debatable topic, we decided to define hypotension based on the primary authors’ / investigators’ definition. We have amended the inclusion criteria by defining isolated hypotension and the clinical and / or biochemical features of hypoperfusion (the clinical / biochemical criteria for defining hypoperfusion was already mentioned in S4Table under the sub-section ‘implementation considerations’).

Intervention(I) (active treatment group): Treatment with inotropes for IH. Volume

expansion with crystalloids or colloids could have been used prior to inotrope or

vasopressor initiation.

Comparator(C) (restrictive treatment group): Treatment with volume expansion and/or inotropes in preterm neonates with hypotension only when clinical and / or biochemical features of poor perfusion were present. The clinical and / or biochemical signs of hypoperfusion were defined as presence of either of these: Unexplained tachycardia (>160-170 beats/min), prolonged capillary refilling time (> 3-4 seconds), low peripheral pulses, decreased urine output (< 1 ml/kg/ hour for 4 -6 hours, physiological oliguria or anuria should also be considered), increasing lactate levels (> 3-4 mmol/L) and base deficit (> 8 meq/L).

b. No treatment of IH. (Page 4, Lines 92 – 100)

As you can see that the infant who are getting fluid bolus might be considered in intervention by some whereas control in another study.

Two arms of study by Pereiera have been combined together as intervention ( active and moderate) when the two are very different. Also by this way they have done "double counting” of the control group which is not appropriate. That total sample size of two RCts is 118 whereas the authors mentioned it as 139 (Table 3 GRADE). It happened at multiple places because of double counting error.

**Response:**

1. We would like to thank the Reviewer for the query. It is to be noted that volume expansion in hypotension is a standard of care in the setting of hypotension in most of NICUs across the globe.
A. The HIP trial protocol states “Infants received an infusion of 10mL/kg of 0.9% saline administered over 20 min before commencing their allocated study drug contained in 50 mL syringes identical in appearance.”
B. Pereira et al. trial states “ A written protocol tailored for each gestation and study arm was at the cot side to guide staff, with BP intervention levels. Apart from this target, the guideline (Appendix A) was similar to standard NICU policy. Key elements included triggers for evaluation (BP, tachycardia, impaired perfusion), basic stabilisation, and a maximum of 10-20 mls/kg intravascular volume.” Henceforth, these two trials had used volume expansion in the intervention and control arms prior to randomisation. Coming to observational studies that were included in the meta-analyses most have not stated whether a bolus had been given or not. So, we are unable to comment on this aspect.
2. We would like to thank the Reviewer for pointing out the aspect of ‘double counting’ related to the Pereira et al. RCT in the meta-analyses of the various outcomes. Of the various approaches to avoid the ‘unit-of-analysis’ error that could happen due to this, we decided to adopt the ‘combining groups’ approach suggested by the Cochrane group ( https://onlinelibrary.wiley.com/doi/full/10.1002/cesm.12033). All the meta-analyses for the various outcomes have been re-done. Figure 2 has been modified. The effect estimates and sample sizes of the various outcomes have been changed in the manuscript. Table 3, the summary of findings table has been changed in accordance with the results of the meta-analyses. All these changes have been marked in the marked copy of the revised manuscript.
3. Many observational studies do not actually meet the criteria for inclusion. They are

hugely different from each other with significant overlap between two groups among

themselves and with other studies.

**Response:**

We thank the Reviewer for the comments. We would like to indicate that this was a systematic review based on which a clinical practice guideline was formulated. Likewise for any clinical practice guideline, we had to adopt a broader inclusion criteria. The specific purpose for the same are two reasons. Firstly, many of these studies would be included in the additional evidence section of the evidence to decision table of the GRADE. I would like to refer to two ILCOR Neonatal Life Support Systematic Reviews

( 1. Ramaswamy VV, Dawson JA, de Almeida MF, Trevisanuto D, Nakwa FL, Kamlin COF, Trang J, Wyckoff MH, Weiner GM, Liley HG; International Liaison Committee on Resuscitation Neonatal Life Support Task Force. Maintaining normothermia immediately after birth in preterm infants <34 weeks' gestation: A systematic review and meta-analysis. Resuscitation. 2023 Oct;191:109934. doi: 10.1016/j.resuscitation.2023.109934. Epub 2023 Aug 18. PMID: 37597649.;

2. Ramaswamy VV, de Almeida MF, Dawson JA, Trevisanuto D, Nakwa FL, Kamlin CO, Hosono S, Wyckoff MH, Liley HG; International Liaison Committee on Resuscitation Neonatal Life Support Task Force. Maintaining normal temperature immediately after birth in late preterm and term infants: A systematic review and meta-analysis. Resuscitation. 2022 Nov;180:81-98. doi: 10.1016/j.resuscitation.2022.09.014. Epub 2022 Sep 27. PMID: 36174764.) where various papers which had not reported on the pre-specified outcomes where included. For example, one paper had evaluated the comfort of doctors and nurses in the OT at different OT temperatures. This was included as an additional evidence in EtD framework. Many QI studies were included where interventions not specified a priori such as education, training etc were components of the QI. To summarise, systematic reviews done for the purpose of formulating CPGs would usually be pragmatic (as it is in this manuscript) while isolated systematic reviews would be more of an explanatory in nature.

3. I think the manuscript definitely have merits but needs a different way of presentation. The statistical analysis seems incorrect to me. It is like combining apples and oranges and then justifying by looking at statistical heterogeneity (while ignoring clinical heterogeneity)

I am getting a feeling that a narrative review without combing the clinically different studies might be more useful. We agree that there are are extremely limited studies on this aspect and but e they are not alike to be combined together.

**Response:**

We thank the Reviewer for the comments. We would like to emphasize on the following points apart from the response provided to Reviewer query 4 (regarding adopting a pragmatic broader inclusion criteria for the systematic review)

(referencehttps://www.sciencedirect.com/science/article/pii/S089543562300046X):

A. The GRADE working group emphasizes on statistical heterogeneity more than clinical heterogeneity which is a norm in pragmatic systematic reviews.
B. The GRADE working group also states the following: “First, if results prove similar from study to study, the variability in study PICOs enhances the generalizability of those results. If patient populations differ widely and results are nevertheless similar across studies, it tells us that patients with different characteristics respond similarly to the intervention, allowing application of the results to a wide range of patients. If studies use different doses but results are nevertheless similar across studies, this tells us intervention effects are independent of dose. Clinicians can then choose freely from among the doses used in the studies (e.g., use lower doses that are less likely to cause adverse effects). Similar interpretations would apply to differences in the comparator and outcomes.” From the perspective of our review, the aforementioned statement may be construed as follows:

- Irrespective of the definition of IH used, the restrictive approach might possibly result in better outcomes.
- These results are generalizable to NICUs where volume expansion alone is used (and not inotropes or vasopressors) for treating IH (restrictive approach).

C. We had already stated this aspect in the limitations section for the sake of transparency as follows:

“There were several limitations to this meta-analysis. The definition of isolated hypotension and clinical hypotension with signs of hypoperfusion varied between studies. Further, some of the neonates in the restrictive approach group received volume expansion which could have altered the effect estimates of the meta-analyses. We tried to address these by downrating the evidence by one level for the domain of indirectness related to patient population and the intervention / comparator.**”**

D. In the previous review, the Reviewer 1 had specifically asked to delete the narrative review section all together which we moved to Appendix in the supplement. Reviewer 1: “I feel the paragraphs on the narrative review may be deleted altogether to improve the readability of the study. However some sections of the narrative review may be used by the authors in the discussion to understand how some the weak or expert recommendations were arrived at.”

Reviewer 1’s response to our edits: “The authors have made all the changes as suggested. The narrative review is included in the appendix. Readability improved. No further changes are recommended”

E. Since we had addressed this issue of this systematic review being predominantly reported based on the results from meta-analyses of various outcomes, we are of the opinion that it would be unfair to make it a narrative review at this stage as the CPG has been widely adopted in our country as well others.
F. Finally, the definition of isolated hypotension did not vary significantly between the studies included in the meta-analyses. The two RCTs had taken almost similar (if not exactly the same definitions). The results of our meta-analyses also did not reveal any statistical heterogeneity as stated before. Hence, we feel that meta-analyses of these two small sized RCTs may possibly be justified. Coming to the observational studies, Faust et al., Aladangady et al., Durrmeyer et al., Kuint et al., and Gogcu et al. have all used quite similar definitions to define hypotension which was MAP < GA. Only Dammann et al. had used a different definition (quartile based). We included Dammann et al. study as we presumed the definition used was quite similar to that of Faust et al.’s as the BP centiles in Dammann et al.’s study were almost similar to MAP < GA. Batton et al. had used a divergent definition for IH and we did not include the study in the meta-analysis. Also, we had addressed this issue by downrating the evidence for indirectness related to definition of IH (population) wherever we deemed appropriate.
